# Chemical Approaches for Structure and Function of RNA in Postgenomic Era

**DOI:** 10.1155/2012/369058

**Published:** 2012-01-12

**Authors:** Tae Suk Ro-Choi, Yong Chun Choi

**Affiliations:** Departments of Pharmacology and Biochemistry, College of Medicine, Dong-A University, 3-1 Dong Dae Shin Dong, Seo Gu, Busan 602-714, Republic of Korea

## Abstract

In the study of cellular RNA chemistry, a major thrust of research focused upon sequence determinations for decades. Structures of snRNAs (4.5S RNA I (Alu), U1, U2, U3, U4, U5, and U6) were determined at Baylor College of Medicine, Houston, Tex, in an earlier time of pregenomic era. They show novel modifications including base methylation, sugar methylation, 5′-cap structures (types 0–III) and sequence heterogeneity. This work offered an exciting problem of posttranscriptional modification and underwent numerous significant advances through technological revolutions during pregenomic, genomic, and postgenomic eras. Presently, snRNA research is making progresses involved in enzymology of snRNA modifications, molecular evolution, mechanism of spliceosome assembly, chemical mechanism of intron removal, high-order structure of snRNA in spliceosome, and pathology of splicing. These works are destined to reach final pathway of work “Function and Structure of Spliceosome” in addition to exciting new exploitation of other noncoding RNAs in all aspects of regulatory functions.

## 1. Introduction

A key element in the study of cellular RNA metabolism is the molecular characterization of RNA. This characterization requires accurate determination of the RNA sequence. It is imperative to understand how RNA structure complements the functional definition of RNA. Cellular RNAs are posttranscriptionally modified at various points in the primary RNA transcript as well as processed. In cellular RNA metabolisms, RNA maturation is performed through various structural alterations that include chemical modifications of constituent components. A most representative modification is observed in chain shortening, rearrangements by transfer of phosphodiester linkages involved in splicing mechanisms (pre-mRNA), deletions (pre-rRNA), and transsplicing (trypanosomal mRNA). Another is chain expansion demonstrated by modifications observed on polyadenylation, U-addition at 3′ ends, 5′-cap formation at 5′ ends, and insertions within trypanosome RNA. Other examples of modifications are base modifications, such as deaminations, methylations, hypermodifications, and ribose methylations.

The most modified RNAs are tRNAs containing approximately 2–22 modified nucleotides per molecule of ~75 nucleotide length, and there have been more than 130 different signature modified nucleotides reported [1]. The discovery of snRNA and m_3_^2.2.7^G caps occurred within the last 50 years. They also contain their own specific modified nucleotides such as Ψ, m^6^A, m^2^G, and 2′-O-methylated nucleotides (Table 1). 

The next class is the ribosomal RNAs which contain 204–209 modified nucleotides within 18S (1,869 nt) + 28S (5,035 nt) RNA in eukaryotes. The mRNAs contain the least modified nucleotides, with the exception of the 5′ end cap structure and occasional m^6^A in the molecule.

In ensuing years, massive scale DNA sequencing was advanced to accommodate the “Human Genome Project.” Two groups published the genomic map where the coding genes were cataloged. It was conservatively estimated that there are 25,000 genes and 50,000 proteomes involved in cell metabolism. It was also envisioned that processing mechanisms could be discerned by comparing the genomic structure with the RNA sequence determined using cDNA methods. Based on the ever-increasing number of RNA sequences, it was determined that most coding RNAs mature as a result of alternative splicing. Aberrant splicing is attributed to point mutations in the genetic code and splicing code [2]. It is noted that RNA sequencing can aid the determination of the molecular pathogenesis of diseases.

## 2. Historical Venture of RNA Research

Detailed nucleic acid chemistry began with discoveries of the DNA helix by Watson and Crick [3] and DNA polymerase by Lehman et al. [4, 5]. With DNA being the genetic material providing a blue print for living creatures, it moved genomic era thinking away from the earlier notion that protein, carbohydrate, and lipid were the only essences of living things.

**Figure 1:**
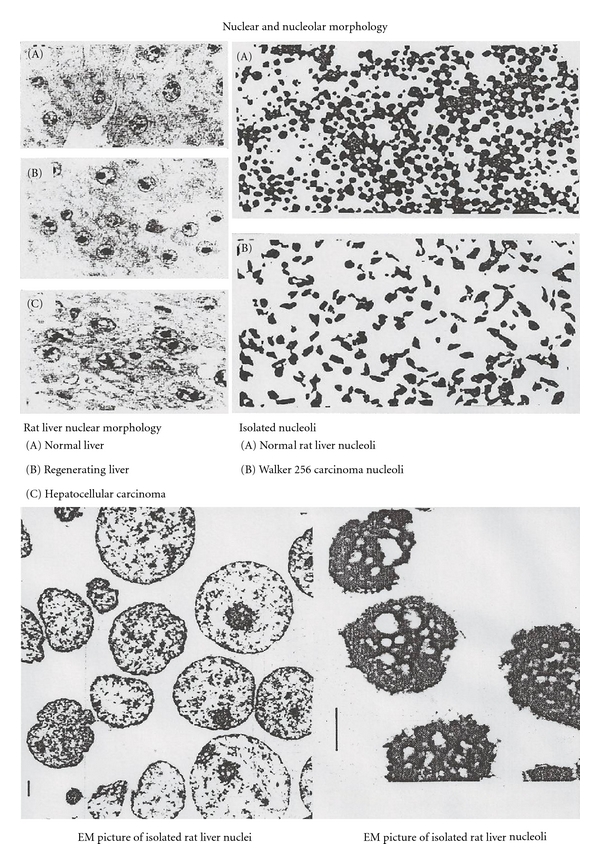
Nuclear and nucleolar morphology. Normal rat liver nuclei have 1–6 round nucleoli which are less than 2 *μ*m in diameter. In regenerating liver, cells contain enlarged nucleoli. In tumor cells (hepatocellular carcinoma), the nucleoli are not only enlarged but also they become pleomorphic in morphology. Nuclei were isolated by homogenization in 2.3–2.4 M sucrose containing 3.3 mM CaCl_2_. Nuclei were sonicated in 0.34 M sucrose and layered on 0.88 M sucrose for purification by centrifugation. Isolated nuclei and nucleoli had high purity, and morphologies were well preserved [6].

DNA is there to provide information needed to build the cells, tissues, organs, and whole individuals. It took a long time to move from the histochemical presentation of DNA in the nucleus and RNA in the nucleolus and cytoplasm [14] to the isolation of nucleoli, nuclei, mitochondria and ribosomes, facilitating the elucidation of their components, their structures, and their functions. Even within the same species, no two individuals are identical. Disarray in DNA structure can determine whether one is healthy or diseased. In the quest to conquer cancer, differences in cellular morphology and uncontrolled growth became and remain a major research consideration when one compares normal cells with cancerous cells and tissues. Cancer cells with pleomorphic, hypertrophic nuclear, and nucleolar morphology remain a useful pathological criterion for a cancer diagnosis. The information within genes is transferred to RNA and then to proteins made on ribosomes that define a cell phenotype. The fractionation of cells into various components includes nucleoli, nuclei (Figure 1), ribosomes, mitochondria, cytosol and others.

The main interest among these compartmental components was the RNA. The RNA has its own exclusive properties which are not found in DNA.

**Figure 2:**
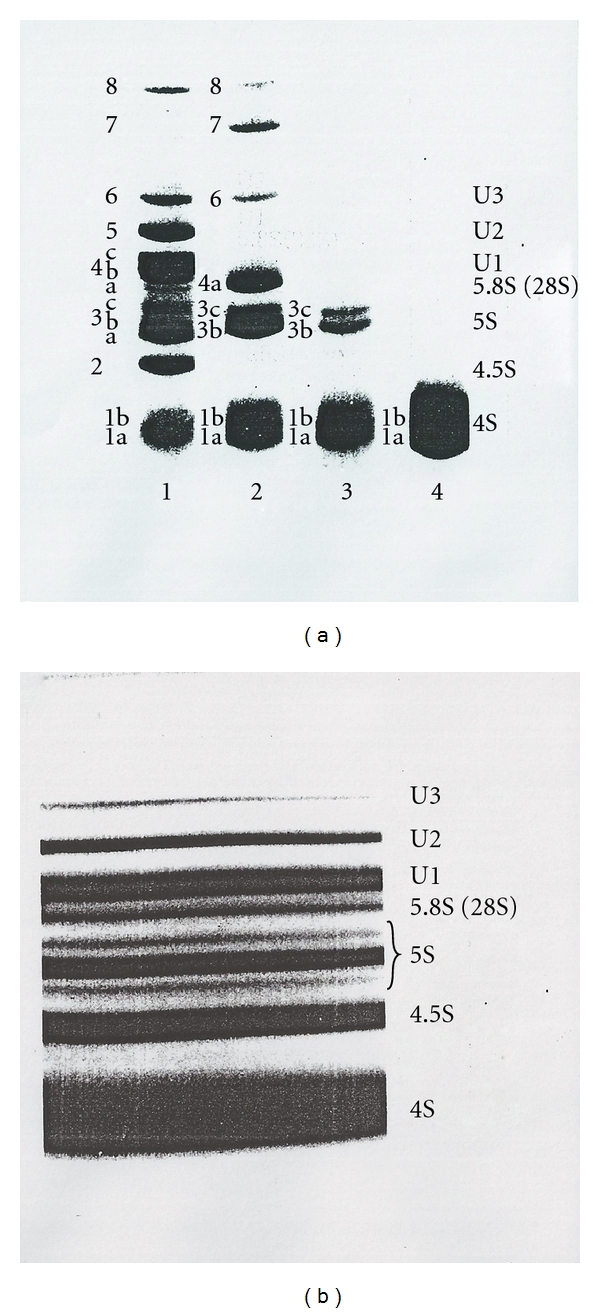
Polyacrylamide gel electrophoretic separations of nuclear 4–7S RNAs of rat liver and Novikoff hepatoma cells [7]. (a) The 8% gel electrophoretic patterns of 4–7S RNA from various cell organelles of rat liver. The gel was stained with methylene blue for RNA visualization. (1) Nuclear 4–7S RNA, (2) ribosomal 4–7S RNA, (3) mitochondrial 4–7S RNA, and (4) soluble cytoplasmic sap 4–7S RNA. (b) The 10% slab polyacrylamide gel electrophoretic separation of [^32^P]-labeled 4–7S RNA from Novikoff hepatoma cell nuclei. The gel was autographed with X-ray film.

The discovery of RNA polymerase I in the nucleoli [31] is the landmark of RNA research in these cellular compartments. It was not until 1968, with the introduction of gel electrophoresis into RNA research [32], that subspecies of 4–8S RNAs could be separated from high-molecular-weight RNAs (>18S RNA). Until then, the 4–8S RNAs were considered as tRNAs and their precursors. Different from the prokaryotic cells, eukaryotic cells were shown to have a variety of small RNAs in their nuclei (Figure 2). These RNAs used to be called LMWN RNA (low-molecular weight nuclear RNA) and now the name is unified as snRNA (small nuclear RNA).

These include U1 RNA, U2 RNA, U3 RNA, (named as such because these RNAs contain a high proportion of uridylic acid), 5S RNA III (U5 RNA), 4.5S RNA I (Alu RNA), 4.5S RNA II (U6), and 4.5S RNA III. All of these snRNA species and many more have been sequenced and their functions elucidated in pre-rRNA processing [33] and pre-mRNA splicing [34, 35].

The most interesting discoveries in the midst of sequencing were the very unusual trimethylguanosine cap structure in U1 RNA (m_3_^2,2,7^GpppAmUmAC), U2 RNA (m_3_^2,2,7^GpppAmUmC), U3 RNA (m_3_^2,2,7^GpppAmA(m)AGC), and 5S RNA III (U5 RNA) (m_3_^2,2,7^GpppAmUmAC) [36]. Afterwards, myriads of cap structures in viral RNA and mRNA were discovered [37]. 

The history of RNA sequence work has occurred in three eras. The pregenomic era was devoted to the small RNAs and commenced with the sequence of large RNAs as technology developed for cDNA synthesis, amplification, cloning, and sequencing. The DNA technology was explosive and paved the way toward establishment of sequence technology not only for RNA and cDNA but also for genomic DNA.

In addition to sequence study, the secondary and tertiary structures have also been determined. A representative study was the crystallographic study of RNA-protein interactions. For example, the most well-worked-out motif is RRM (RNA recognition motif) which is most abundant in hnRNP [40] and splicing factors [41]. The summary of characteristics of RRM is in Table 2. 

It has been known for a long time that pre-mRNA (hnRNA) is cotranscriptionally assembled into beads on a string consisting of 30–50S (20–30 nm) particles [42]. The RNP (hnRNP) has usually 48 hnRNP proteins and ~700–800 nucleotide long RNA string [43]. More recently, most hnRNP proteins have been found to have 1-2 RRM motifs for RNA binding. From these characteristics, the primary RNA transcripts have been folded from the 5′ end with the following rules: a minimum of 3 nucleotides in the loop and a minimum of 3 base pairs at the stem. According to stacking and loop energy rules, two nucleotide loops cannot exist. The number of base pairs needed for stabilization with the most stable stacking energies by CCC/GGG or GGG/CCC is 3 base pairs with −9.8 kcal and the highest loop destabilizing energy is +8.4 kcal [44]. In addition, protein binding to RNA has been shown to have −∆G *≈* 10−13 Kcal/mol [45] which can overcome the loop destabilizing energies of any size. With this rule, folding the hnRNA in GC, AU, and GU pairings was carried out as the RNA was transcribed, extending contiguous base pairing until it comes to a base pair mismatches. Accordingly, small simple RNA hairpins have been constructed with the aid of a computer [46] from the 5′ end (transcription start sites). Consensus patterns for folding characteristics have been observed (Table 3).

**Figure 3:**
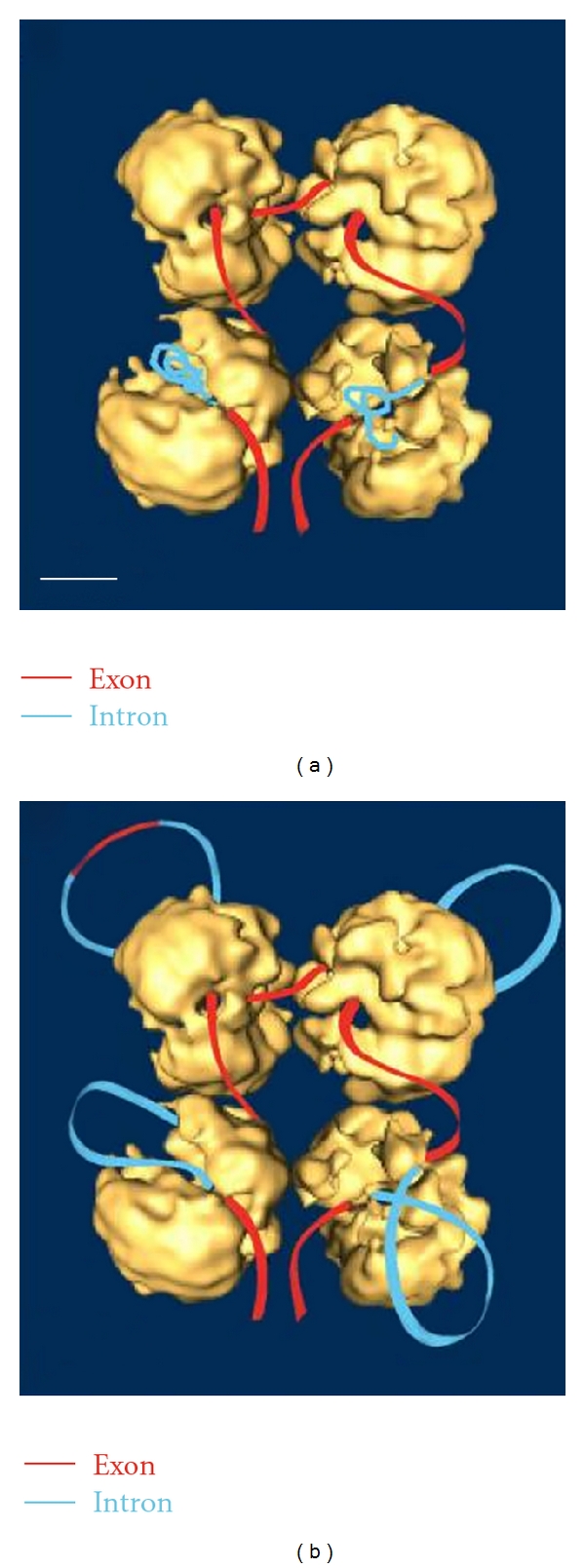
The supraspliceosome model from the article by Sperling et al. [8]. (a) It was stated that pre-mRNA which is not being processed is folded and protected within the native spliceosome. (b) With different staining protocol, it was possible to visualize the RNA strands and loops emanating from the supraspliceosome. These complexes were found to contain hnRNP proteins (personal communication).

The transcripts form one stem loop for every 15–18 nucleotides which is consistent with ~15–17 nucleotides per hnRNP protein (700–800 nucleotides per 48 hnRNPs in one hnRNP particle) reported earlier [43]. The thermodynamics of RNA folding was consistent with the order of splicing in ovomucoid pre-mRNA [47]. From the point of view that supraspliceosomes contain hnRNP proteins (personal communication), it may be that this cotranscriptional formation of hnRNP string particles [47–49] may contribute to a role in the formation of supraspliceosomal RNP (Figure 3) [8].

The postgenomic era is the present day era or the second generation genome era. With the recent discovery that there is a paradox [50, 51] in the cellular transcript number, which is 2-3-fold in excess and that 50% of the cellular transcripts are ncRNAs, the second generation genomic era is in the process of resequencing the genome for ncRNAs. It is anticipated that there will be a revision in the first generation genomic picture. In this era, work is proceeding that will probe and dissect the RNA metabolism in which aberrant processing should be elucidated by RNA sequencing. To dissect the molecular pathology of RNA metabolism, it is also necessary to study higher-order structures based on the sequence studies involved in the assembly of macromolecular machinery. It is natural to hope that therapeutic interventions will be discovered that can correct errors in the genetic code and its product splicing. 

The RNAs have been classified according to the following diverse basis of criteria:

- cell biology: cell types, subcellular origins,
- molecular weight: high molecular weight (HMW) and low molecular weight (LMW/small),
- S value: 5S rRNA, 7S RNA, 18S RNA, and others,
- linearity: linear, cyclized, and branched (Y shaped),
- metabolism: precursor, processed intermediates, and mature,
- standard: hnRNA, rRNA, mRNA, tRNA, and ncRNA (snRNA, snoRNA, miRNA, and others as in Table 4).

## 3. Preparation of RNA from Isolated Subcellular Compartments

RNA can be extracted from purified nucleoli, nuclei, ribosomes, mitochondria, and cytosol by the SDS-phenol procedure. The procedure involves the suspension of organelles in 0.3–0.5% SDS (sodium dodecyl sulfate), 0.14 M NaCl, and 0.05 M sodium acetate buffer at pH 5.0 and deproteinization by phenol containing 0.1% 8-hydroxyquinoline at 65°C [53]. The extracted RNA is precipitated with 2–2.5 volumes of ethanol containing 2% potassium acetate. The RNA is washed by ethanol and dissolved in appropriate buffer for the analysis. The DNA and protein contaminations are less than 3% by weight. The purified RNA is separated into individual RNA species using sucrose density gradient centrifugation, gel electrophoresis, and column chromatography [38].

## 4. Structure Determination

### 4.1. Structural Characteristics of Various RNAs Bearing Signature Sequences and Modifications

The RNA is composed of basic 4 nucleosides of guanosine, adenosine, uridine, and cytidine linked by 5′-3′ phosphodiester bonds between two ribose moieties. In addition, some of these nucleotides are modified in base as well as in ribose moieties and contain unusual pyrophosphate bonds at their 5′ ends and 2′ O-methylated 3′ end.

**Figure 4:**
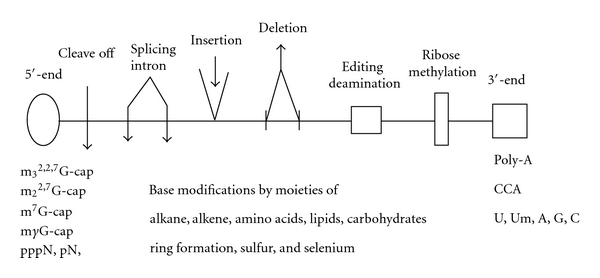
Summary of RNA modifications. Cotranscriptional and posttranscriptional RNA modifications are summarized.

Mature RNAs are synthesized in the nuclei and directed by the posttranscriptional processing machineries. Because of these specific modifications, there is a general consensus on the presence of specific signature sequences and modifications for the identity of RNA classes. Based on extensive sequence work, it is possible to classify RNAs according to structural modifications.  Figure 4 provides an outline for characteristics of RNA, and its modifications and brief examples are given in Table 5.

### 4.2. General Scheme of RNA Sequencing

The very first RNA sequence was obtained from the work of yeast alanine tRNA in 1965 [54]. In this work, the prerequisites for RNA sequence work were developed and described. Since then, it is a fundamental approach to establish oligonucleotide catalogs using specific RNases. One set is the catalog of T1 oligonucleotides produced by RNase T1. The other is the catalog of oligonucleotides produced by RNase A. The analytical method was based on UV spectral absorption in the earlier years. Subsequently, since 1970, isotopic labeling methods were widely used which are 1,000-fold more sensitive. Furthermore, many other improvements in RNA sequence technique have made it possible to advance the rate of RNA sequence work greatly (Table 6). 

Improvement was observed in the following areas: (1) RNA labeling techniques, (2) fractionation procedures (chromatography, electrophoresis, and gel procedures), (3) use of various RNases, (4) contig seeking, and (5) ladder sequence gel analysis. For example, based on labeling at the 5′-end with [^32^P]-*γ*-ATP by polynucleotide kinase [56], it has become feasible to read a 150 nucleotide sequence using an endonuclease assisted ladder gel from the 3′-end. Also, based on labeling at the 3′-end with [^32^P]-5′-pCp by RNA ligase [57], it has become feasible to read approximately 150 nucleotides from the 5′-end. Together, these enhancements make it readily feasible to sequence RNA with approximately 300 nucleotides. In contrast to success in the sequence work for small RNAs, two challenges remained. One challenge is related to RNA size and the other is concerned with scarce abundance of RNA in the cell. With the discovery of reverse transcriptase, heat stable DNA polymerase, and recombinant technology, it became possible to produce cDNA, amplify, and clone by RT-PCR methods.

With high-efficiency RT-PCR, high-molecular-weight RNA with 10,000 nucleotides in length can be readily sequenced [59]. A remaining shortcoming of this approach is the inability to fully characterize modified nucleotides. However, ability to deal with long chain lengths and scarce abundance outweighs this limitation. cDNA-based methods clearly dominate any RNA sequence work that involves long RNA length or low RNA abundance. Examples are observed in the direct gene isolation for cleavage controlled processing RNAs (Pre-rRNA and rRNA) and cDNA method for pre-mRNA and mRNAs. Therefore, as a result of accumulated methodologies, it becomes common that RNA sequence can be obtained through more than one scheme or type of technique, such as straight chemical approaches [60] or biotechnology-mediated approaches.

### 4.3. Outlined Steps of Sequence Work

Brief outlines are described for sequencing RNAs. It may be divided into two methods although combined methodology is in fact feasible. 

#### 4.3.1. Direct Method of RNA Sequencing

**(a) Preliminary Examination of External Glycol Structures**

In some cases, a rapid diagnostic examination is required. Most convenient procedures employ the use of specific antibodies against different forms of 5′-cap structure (m^7^G cap or m_3_^2,2,7^G cap) and a oligo-dT column for poly-A affinity chromatography. Alternatively, a [^3^H]-derivative method can be useful. The radioactive labeling of terminals was performed using the periodate oxidation method, followed by reduction with [^3^H]-borohydride. T_2_ RNase digestion and fractionation by paper chromatography reveal the presence of the 3′-terminal and 5′-cap.

**(b) Selection of Labeling Methods**

RNA can be labeled in vivo (prelabeling) or in vitro (postlabeling).

In vivo labeling is carried out by incubation of living cells in the presence of [^32^P]-phosphate in a phosphate-free medium. RNA is uniformly labeled by this method.

In vitro labeling is called postlabeling because it labels the isolated RNA with isotopic agents such as [^32^P]-phosphate or [^3^H]-borohydride. [^32^P]-labeling can be carried out using kinase enzymes. The 5′-labeling is done with [^32^P]-ATP by polynucleotide kinase, that is, provided the 5′-end is free from phosphate. If the 5′-end is blocked by the presence of a 5′-cap structure, the pyrophosphate moiety must be removed by a pyrophosphatase and phosphatase. And then the kinase method can be employed to introduce the tracer. Labeling at the 3′-end is done with [^32^P]-pCp by RNA ligase. The [^3^H]-derivative (nucleotide diol) with [^3^H]-borohydride indicates that the 3′-end is free from phosphate or any other blocking structures. A shortcoming of [^32^P]-labeling is the short half-life of the isotope which provides a working period of approximately 4 half-lives. The main limitation of the [^3^H]-labeling method is weak energy of the tritium isotope. This can make the reading of the autoradiograph for a ladder sequencing gel very difficult.

**(c) Initial Reading of Sequence by Ladder Sequencing Gel**

To obtain the nucleotide sequence of RNA quickly without characterization of modified nucleotides, it is common to use the endonucleases-dependent sequencing technique [61]. Terminal labeled RNA (5′-end or 3′-end) is partially digested with specific endonucleases (T_1_, U_2_, A, phys I, and others), and each product is loaded in parallel on a 10–15% denaturing polyacrylamide gel. Note that if crude acrylamide is used, the running temperature of the gel can quickly rise to 60–70°C. Since the mode of cleavage is known, it is possible to discern G (T_1_), A (U_2_), U and C (A) and C-resistance (Phys I). It is not uncommon to read an RNA sequence using this method within one day.

**(d) Base Composition**

There are two technical approaches that can be used to determine RNA base composition (levels of nucleotides or of nucleosides).

RNase T_2 _ or alkali (0.3 N KOH) is used to complete hydrolysis. But alkali (0.3 N K/NaOH) is not preferred because it destroys 7-methyl purines. Prelabeled [^32^P]-RNA is hydrolyzed, and its products are separated by 2-dimensional paper chromatography followed by autoradiography [62]. Since the standard separation pattern is known, various modified nucleotides are readily identified by comparison [56].

**Figure 5:**
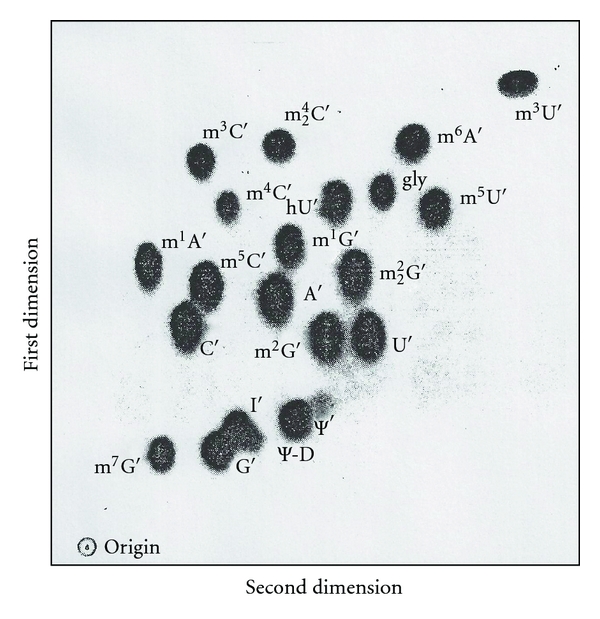
Two-dimensional map of standard nucleosides [9]. The [^3^H]-labeled standard nucleoside derivatives are separated by two-dimensional thin layer chromatography. The first dimension is shown from bottom to top and the second dimension from left to right. Solvent systems are in the text. N′ represents trialcohol derivatives of representative nucleosides.

Alternatively, after cold RNA is digested into constituent nucleotides, which are subsequently dephosphorylated by phosphatase, the resulting nucleosides are converted into [^3^H]-derivatives and separated by thin layer chromatography. The separated nucleosides (including all modified nucleosides except 2′-O-methylated nucleosides) are detected by fluorography and identified based upon a standard migration pattern (Figure 5) [9].

**(e) Catalogs of Oligonucleotides**

Two types of catalogs are made. One is an RNase T_1_ catalog, and the other is an RNase A catalog.

To map oligonucleotides, two necessary procedures are essential. The first is to prepare labeled oligonucleotides and the second is to fractionate two-dimensionally.

To obtain labeled oligonucleotides, three approaches are possible.

1. Use of prelabeled [^32^P]-RNA for specific endonuclease digestion.
2. 5′ labeling after enzyme digestion using [^32^P]ATP and polynucleotide kinase.
3. 3′ labeling after endonuclease digestion and removal of resultant 3′-phosphate by phosphatase. Then the labeled derivatives can be formed by [^32^P]-5′-pCp and RNA ligase or periodate oxidation followed by [^3^H]-borohydride reduction.

**To Map Oligonucleotides**

There are a number of different techniques. However, the most common are a combination of high voltage paper electrophoresis on cellulose acetate at pH 3.5 and high voltage DEAE paper electrophoresis (7% formic acid) or high voltage electrophoresis on cellulose acetate at pH 3.5 followed by DEAE homochromatography at 60–70°C. Another method that can be used is two-dimensional thin layer (PEI) chromatography using two-solvent systems [63]. Detection is performed by autoradiography. It is notable that T1 oligonucleotides from 45S pre-rRNA can be fractionated into approximately 200 spots by homochromatography [64].

**To Sequence Oligonucleotides**

Several enzymatic digestions can be exploited.

The recovered [^32^P]-oligonucleotides (prelabeled) are subjected to secondary digestions with RNase U_2_ for placement of A residues, RNase T_1_ for G residues, RNase A for U, and C residues plus other endonucleases. Treatment with exonucleases (spleen phosphodiesterase, snake venom phosphodiesterase), and partial digestion with the enzymes above is required to sequence RNA. In each step, nucleotide composition is determined.

***To Determine the Sequence of *5′*-Labeled [^32^P]-Oligo-Nucleotides***

A mobility shift test can be applied [56]. After partial hydrolysis with snake venom phosphodiesterase the product is fractionated by homochromatography or PEI thin layer chromatography. The mobility shift pattern is produced according to the step-wise loss of each nucleotide from the 3′-end. The resulting pattern can be used to read the sequence of the oligonucleotides.

**To Determine the Sequence of [^3^H]-Oligonucleotides**

The procedures used for prelabeled [^32^P]-oligonucleotides are applicable. Secondary digestion methods and accompanying [^3^H]-derivative methods for the determination of nucleotide composition can be carried out.

It may be necessary to strengthen the catalog of oligonucleotides. Generally this involves the expansion of the catalog to provide contiguous overlapping sequences. A feasible approach is to produce large fragments (purified on 10–15% denaturing polyacrylamide gel electrophoresis) and identify the overlapping oligonucleotides. Usually a limited fragmentation by a diluted endonuclease at low temperature or water hydrolysis may produce large overlapping fragments [63]. Examination of large fragments, as done above for ladder gel sequencing and catalogs, can often clarify any ambiguity encountered. An excellent example of one hit hydrolysis is observed in the work on tRNA structure [63]. Based on these very same methods, it can be summarized that many small RNAs have been sequenced. These include tRNAs, pre-tRNAs, 4.5S RNA I, 5S rRNA, 5.8S rRNA, snRNAs, snoRNAs, 7S RNA, and some fragments of pre-rRNA, 28S rRNA, and 18S rRNA. 

#### 4.3.2. Indirect Method of RNA Sequencing

The indirect method of RNA sequencing using cDNA or DNA gene analysis was developed as part of explosive advancements with DNA biotechnology. The direct RNA sequencing method proved useful for the characterization of small RNAs (~100–300 nt). However, sequencing high-molecular-weight (HMW) RNAs proved to be too difficult. Moreover, HMW RNAs that are scarce abundance often do not meet the sample amounts required by the former methods. The search for a solution to this dilemma was successful. One solution involved the isolation of the gene that codes for a specific RNA and the other is to synthesize cDNA which can also be used to isolate a specific RNA gene. Using DNA biotechnology, it proved possible to scale up and solve “The Human Genome Project.” Several genomes have been sequenced, specifically the human (2.9 Gb) and mouse (2.5 Gb) genomes [65–67]. In well equipped laboratories, it is possible to sequence DNA at the rate of 10^6^–10^7^ nt/day. This technology has been widely commercialized and is currently available as kits for cDNA cloning, sequencing, along with enzymes and equipment that supports automatic sequencing. The principal objective of the genomic approach was to determine the sequences of the coding genes. Vast collections of sequence data were compiled for RNAs, cDNAs, and genomic structures, revealing the base sequences for a number of RNAs. As a result of this work.

- Unidentified proteins have been predicted to number 25,243; whereas the known protein number is 15,337.
- A majority of mRNA species (95%) mature through alternative splicing mechanisms.
- Disease genes are estimated to be 2,577 in number.
- Point mutations are 31,250 in number; half of disease-causing mutations are attributed to aberrant splicing (disruption of splicing codes) whereas other forms of mutation include disruption of the genetic code.
- Disruption of splicing code occurs at the splice site and enhancer/silencer sites of exonic and intronic sequences.
- Pathogenic sequences that occur as a result of splice code mutations (transition and transversion) cause aberrant modifications of a variety of RNAs [68, 69].

Recently, evidence has been accumulating that suggests a need to revise earlier estimates of the number of transcriptional products arising from the genomic information. Paradoxical findings were obtained that contradicted earlier and more conservative estimates of the proteasomes size (50,000), in fact, the cellular transcripts are 2-3 times higher than estimated earlier [50, 51]. Also, 50% of the transcripts were comprised of noncoding RNA, some of which are polyadenylated. This paradoxical manifestation has led to the second generation of genomic work, strictly based on RNA characterization. It is worth emphasizing that this has become the second genomic frontier where a reevaluation of the first genomic work is necessary. The present task is more daunting than the “The first Generation Genome Project.” The task at hand is to resequence the genome and then categorize and catalogue the ncRNA species by utilizing all available sequence means, including direct sequencing and DNA microarray techniques.

The next step is to construct secondary structures according to enzyme susceptibility and computer-aided base pairing. Interacting proteins will need to be defined by biochemical, NMR, X-ray, and cryo-EM methods.

## 5. Reagent and Procedures Required for Sequencing

### 5.1. RNA-Specific Cleavage Reactions (2′-OH Required Reaction)

1. Mild alkaline hydrolysis (0.3 N KOH) produces 3′ monophosphorylated nucleotides.
2. T_1_ RNase cleaves phosphodiester bonds after G base producing 3′ GMP at the 3′ ends.
3. RNase A cleaves phosphodiester bonds after pyrimidines (U and C) producing 3′ phosphates at 3′ ends.
4. T_2_ RNase cleaves all phosphodiester bonds with a preference for A residues, producing 3′ monophosphates.
5. U_2_ RNase cleaves phosphodiester bonds after A base, producing 3′ monophosphates.

**Figure 6:**
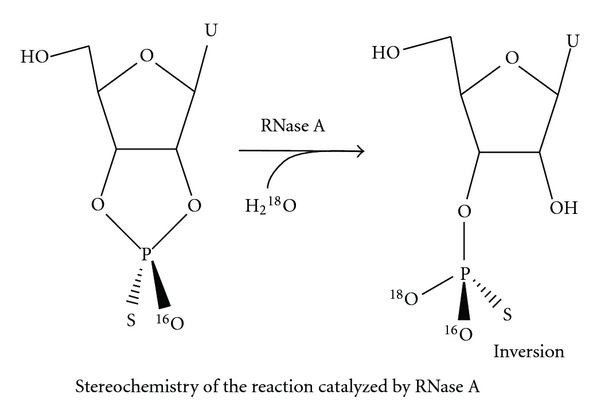
Stereochemistry of the reaction catalyzed by RNase A [10]. The intermediary 2′,3′ cyclic nucleotide (cNp or cNMP) is hydrolyzed to a 3′ phosphorylated mononucleotide. Other 2′-OH requiring enzymatic and alkaline hydrolysis may go through the same path.

The mechanism catalyzed by alkaline hydrolysis, RNase A, T_1_ RNase, T_2_ RNase and U_2_ RNase involves a S_N_2(p) mechanism attacking 2′-hydroxyl groups on the adjacent internucleotidic phosphodiester bond to displace the 5′-hydroxyl group of the neighboring nucleotides and generate a 2′,  3′-cyclic nucleotide intermediate. A subsequent hydrolysis of the 2′,  3′-cyclic nucleotide yields a final product, a 3′ mononucleotide (Figure 6).

### 5.2. The Enzymes Cleaving All Phosphodiester Bonds Including 2′-O-Methylated Ribose

1. P_1_ RNase: the enzymatic digestion by P_1_ RNase cleaves all phosphodiester bonds (except pyrophosphate linkages), producing 5′ monophosphorylated nucleotides.
2. The enzymes acting from the ends for sequencing fragments
  - Snake venom phosphodiesterase (phosphodiesterase I) cleaves phosphodiester bonds, as well as pyrophosphate bonds producing 5′ monophosphorylated nucleotides. It cleaves single-stranded RNA or DNA from the 3′ end in a progressive manner.
  - Spleen phosphodiesterase (phosphodiesterase II) produces 3′ monophosphorylated nucleotides cleaving from nonphosphorylated 5′ ends of single-stranded RNA or DNA.

### 5.3. Other Enzymes Utilized for Sequencing

1. Alkaline phosphatase removes phosphate from 3′ and 5′ ribose moieties.
2. Pyrophosphatase will only cleave pyrophosphate linkages. There are pyrophosphatases from tobacco and potato as well as from *Crotalus adamanteus* venom type II.
3. Using varying combinations of fragmentation methods, it becomes possible to obtain fragments that range in size from nucleosides to very large fragments.

### 5.4. Chemical Modifications Used for Sequencing

#### 5.4.1. CMCT Reaction

**Figure 7:**
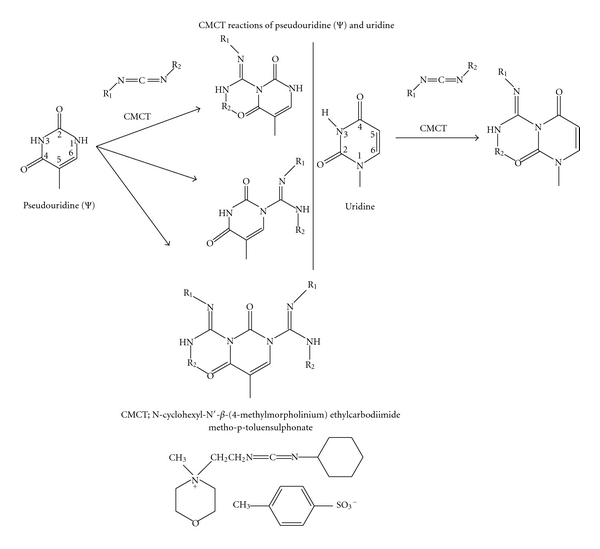
The CMCT reaction of pseudouridine (Ψ) and uridine, and the structure of CMCT [11]. Adducts formed with CMCT on Ψ and U are shown. This adduct formation prevents the cleavage by RNase A at U but not at C. The mild alkaline treatment of reaction products destroys the U but not the Ψ. These differences were utilized to locate the position of Ψ by reverse transcriptase.

Originally reported by Gilham [73], the adduct formation of uridine and guanosine components of RNA with CMCT made uridine residues resistant to RNase A. In addition it has been shown that CMCT reacts with pseudouridine and to a lesser extent with inosine. This reaction takes place on Ψ(N1,N3), U(N3), G(N1), and I(N1), and cold dilute ammonia removes the adducts from Ψ(N1) and hot concentrated ammonia removes remaining adducts from Ψ(N3) [74, 75]. These properties have been used to block RNase A digestion at U but not at C as well as to differentiate U from Ψ (Figure 7) [11].

Direct chemical methods for sequencing RNA using dimethyl sulfate, diethyl pyrocarbonate, and hydrazine followed by aniline-*β*-elimination have been successfully utilized in 5S RNA and 5.8S RNA sequence analysis [60].

#### 5.4.2. DMS (Dimethylsulfate)

This has been used to identify secondary structures as well as for the synthesis of standard m_3_^2,2,7^G. The properties of DMS modifying adenosine (N1) and cytosine (N3) make modified nucleotides unable to base-pair. For this reason RT-PCR stops one nucleotide before the modified nucleotide enabling the location of a modified nucleotide as well as differentiating the single-stranded from double-stranded regions of RNA. DMS also has been used for synthesis of m_3_^2,2,7^G from N2,N2-dimethylguanosine. For this synthesis, the reaction has been carried out by the methods of Saponara and Enger [76]. Twenty milligrams of N2,N2-dimethylguanosine were suspended in 400 *μ*L of dimethylacetamide containing 10 *μ*L dimethylsulfate. The mixture was shaken for 15 hours at room temperature and then centrifuged to remove insoluble products. The supernatant was adjusted to pH 8.0 with concentrated ammonia and then placed on a phosphocellulose column (1 × 50 cm) at pH 7.0 (0.001 M ammonium acetate). A linear gradient of 0.001–0.3 M ammonium acetate was used to elute the samples. One major peak of the product (m_3_^2,2,7^ trimethylguanosine) was found between two minor peaks (corresponding to N2,N2-dimethylguanosine and 7-methylguanosine). The product was lyophilized and identified as m_3_^2,2,7^G by mass spectrometry [12]. The summary of reagent and procedures required for sequencing is provided in Table 7.

**Figure 8:**
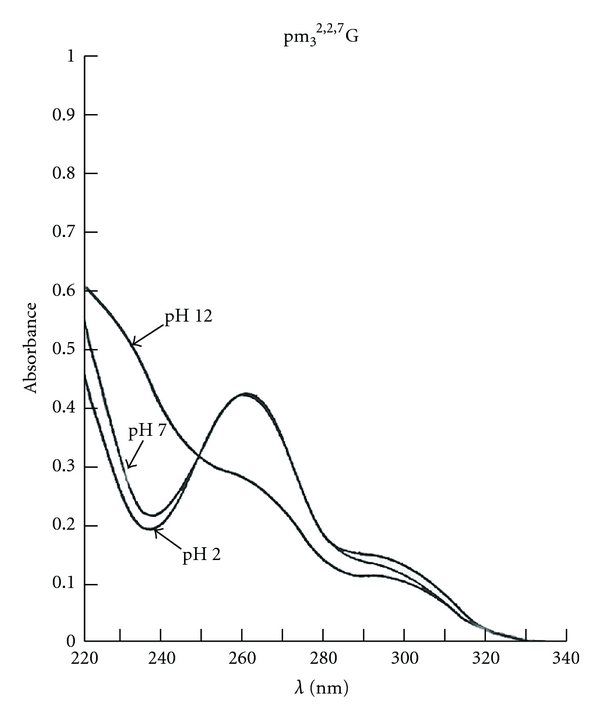
The UV spectra of pm_3_^2,2,7^G [12]. The ultraviolet absorption spectra were recorded on a Cary 14 spectrometer immediately after addition of compound to solutions at pH 2, 7, and 12.

**Figure 9:**
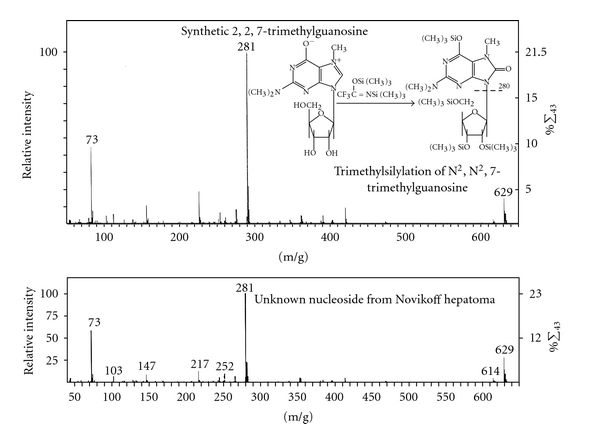
The mass spectra of trimethylguanosine [12]. The synthetic m_3_^2,2,7^G and unknown nucleoside from U2 RNA were trimethylsilylated and subjected to LKB 9000 gas chromatograph-mass spectrometer. The mass spectrum of the unknown nucleoside from U2 RNA was identical to synthetic m_3_^2,2,7^G.

The nucleotides or nucleosides obtained can be separated by column chromatography, paper electrophoresis or thin layer chromatography to determine the number of G, A. U, C and modified residues in the fragments or in the molecule. These 4 bases have specific UV spectra and chemical reactivity to identify the nature of the bases in comparison with known standards. The unusual nucleoside, trimethylguanosine, has its specific UV absorption spectra (Figure 8) and mass spectrometric characteristics (Figure 9).

## 6. The Major snRNA Sequenced

**Figure 10:**
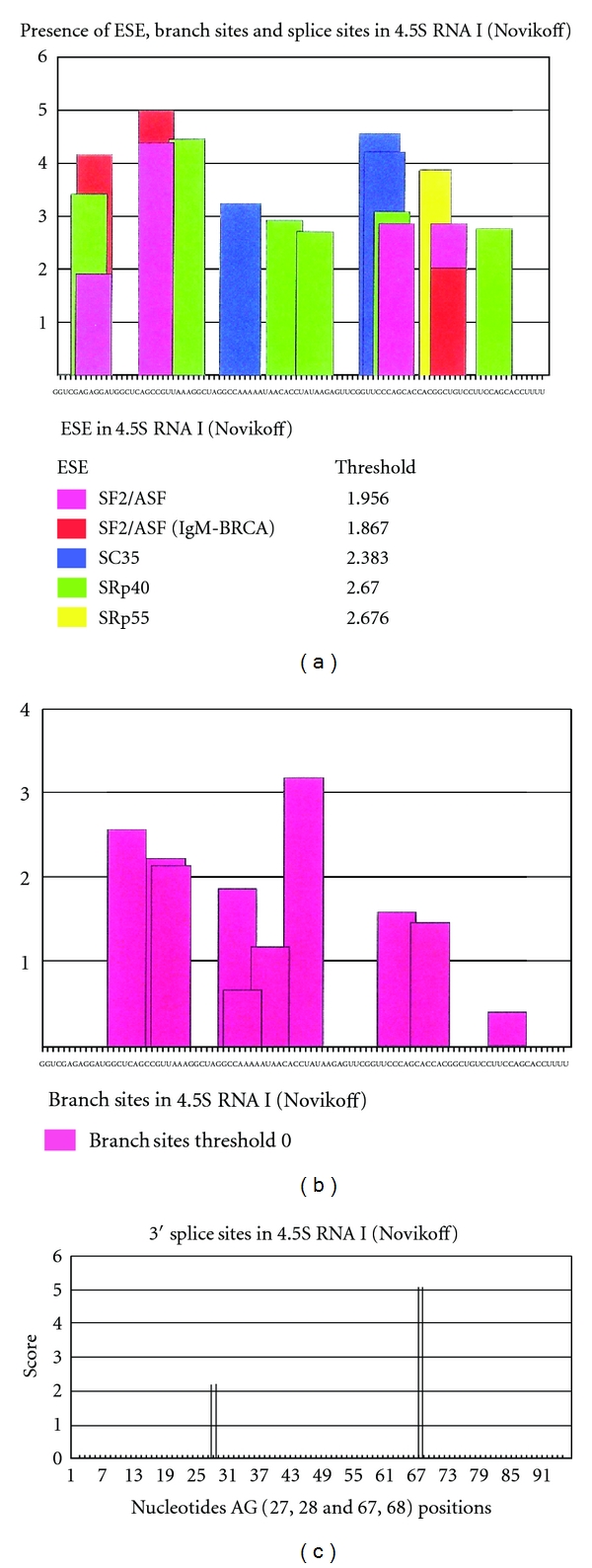
ESE motifs in 4.5S RNA. The sequences of 4.5S RNA I from Novikoff hepatoma cell nuclei were screened by ESE finder 3 [13] for ESE, 5′ splice sites, branch sites, and 3′ splice sites. The default threshold value was used. There were 4 SF2/ASF sites, 3 SC35 sites, 6 SRp40 sites, 1 SRp55 sites, 10 branch sites, and 2 3′ splice sites. These numbers resemble the number identified in Alu elements of human FMR1 transcript (Table 8).

The first nuclear small RNA sequenced was 4.5S RNAI [77] shown in Figure 37. This RNA contains the RNA polymerase III promoter box A and box B like motifs and shows interesting enhancer motif elements resembling the Alu element transcript. The RNA polymerase III promoter areas are underlined and the first nucleotide of the enhancer motif is marked by colored letters. The red color is SF2/ASF (4 motifs), blue color is SC35 (3 motifs), green color is SRp40 (6 motifs), and yellow color is SRp55 (1 motif) (Figure 10(a)). It also exhibits 3′-splice sites marked by [AG] as well as branch sites with the highest score marked by {CACCUAU} (Figure 10(b)). The ESE (exonic splice enhancer), splice sites (Figure 10(c)), and branch sites were examined by ESEfinder 3.0 [13].

In comparison with known Alu elements in the FMR1 gene, the resemblance of 4.5S RNA I in ESE, 5′SS, BS, and 3′SS distribution (Table 8) suggests that 4.5S RNA I is more likely derived from an Alu gene expressed in Novikoff hepatoma cells.

The Alu element has been shown to have many different functions in transcription, splicing, exonization [78], gene insertions (transposons), and DNA replication. It is interesting to observe that the (+) oriented Alu has more 5′ splice sites and the (−) oriented Alu has more 3′ splice sites. It may suggest that exonization may occur from the 5′ side of (+) Alu elements and 3′ side of (−) Alu elements. The SRP RNA (7SL RNA) has Alu elements in its sequence [79]. Whether the Alu is derived from 7SL or Alu is exonized to 7SL is not clear. Subsequently, other snRNAs have been sequenced.

**Figure 11:**
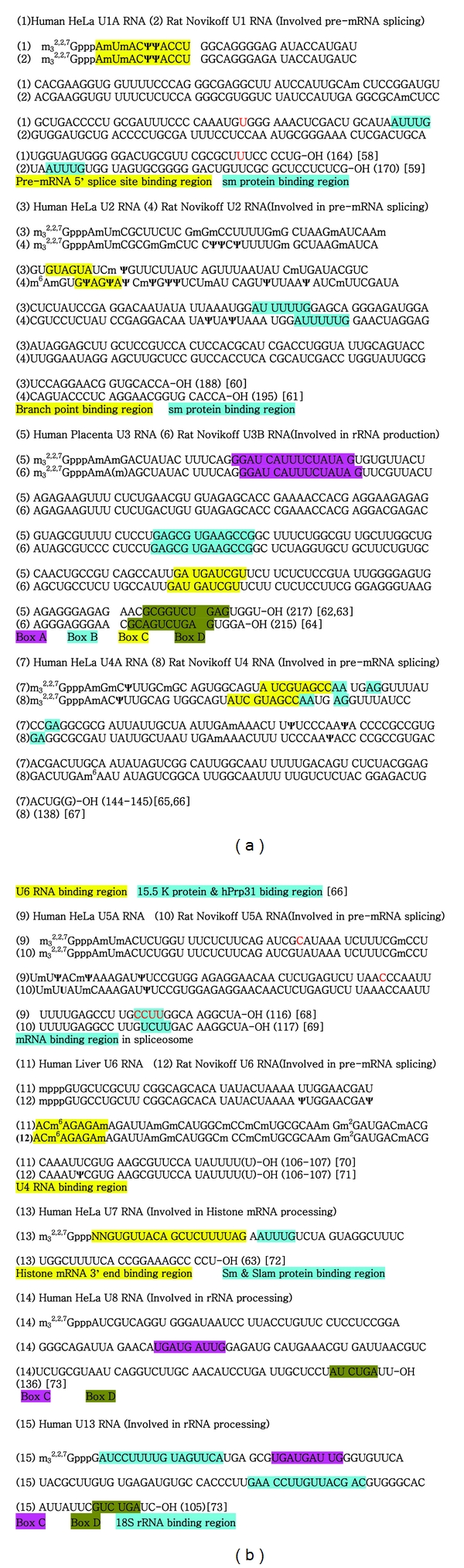
Sequences of major snRNAs (see [15–30]). The sequences of major snRNAs from human and rat involved in splicing and processing are aligned for comparison. The sequence elements in major spliceosomal snRNAs and processosomal snoRNAs are highlighted in the sequences. Those are the pivotal motifs for the function. The numbers in parenthesis are the chain length of the RNAs.

The sequences of the capped snRNAs are described in Figure 11. The pivotal sequences needed for functions are marked by colors.

In the course of any sequence work, there are always challenges in resolving unknown structures at the 5′ end portions which contain the 5′-cap structure and various modified nucleotides. The experimental steps required to discern this complicated region are described.

## 7. Nucleotide Composition and Modified Nucleotides in snRNAs

The compositional analyses were carried out by UV analysis as well as isotope labeling analysis. For example, UV analysis required ~10 mg of U2 RNA. 

### 7.1. RNA Terminal Labeling with [^3^H]-KBH_4_

**Figure 12:**
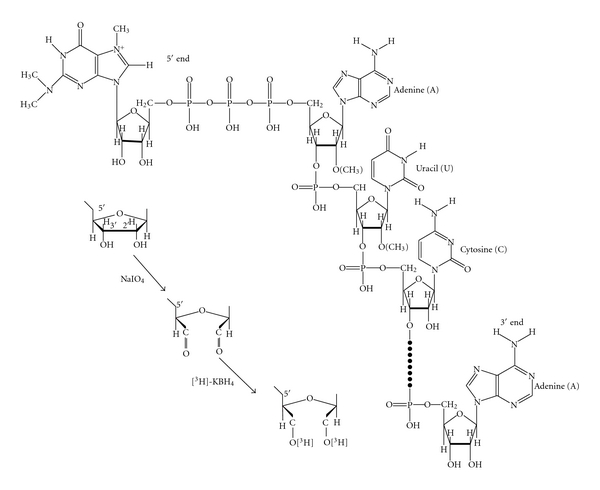
RNA 5′ and 3′ end labeling with [^3^H] by treatment with NaIO_4_ and [^3^H]-KBH_4_. The 4–7S RNA from Novikoff hepatoma cell nuclei was labeled to detect the presence of free 2′-OH and 3′-OH by NaIO_4_ oxidation followed by [^3^H]-KBH_4 _reduction. The reaction occurred at both ends of RNA (5′ end and 3′ end).

The purified nuclear RNAs were separated by sucrose gradient centrifugation which separates 4–8S RNA, 18S RNA, 28S RNA, 35S RNA, and 45S RNA isolated from nuclei of rat liver, Walker tumor, or Novikoff hepatoma cells. As an initial step for the structural characterization, 3′ end nucleosides were labeled by the procedure of sodium periodate (NaIO_4_) oxidation and potassium borohydride ([^3^H]-KBH_4_) reduction. The reaction was carried out in 0.1 M sodium acetate buffer at pH 5 with freshly prepared NaIO_4_ in the dark for 1 hour and precipitated the RNA with ethanol. The RNA was redissolved in the same buffer and treated with ethylene glycol to destroy excess NaIO_4_. The RNA was precipitated with ethanol and redissolved in 0.1 M sodium phosphate buffer, pH 7.7, and treated with radioactive [^3^H]-KBH_4_ [38]. These reaction products would have tritium labeling in cis-alcohols from cis-aldehyde oxidation products of the 2′ and 3′ hydroxyls of ribose, assuming all 3′ ends of RNA have accessible 2′ and 3′ OH groups (Figure 12).

**Figure 13:**
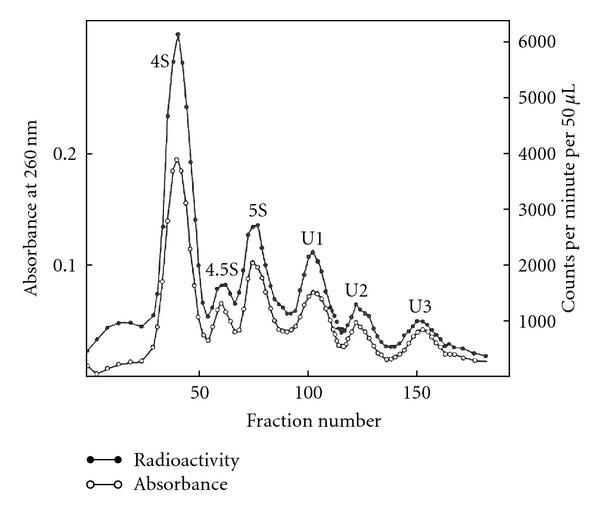
Preparative gel electrophoretic pattern of 4–7S RNA labeled with [^3^H] [16]. The intact, labeled RNA (as in Figure 12) was subjected to preparative polyacrylamide gel electrophoresis. The UV absorption and radioactivity were measured.

**Figure 14:**
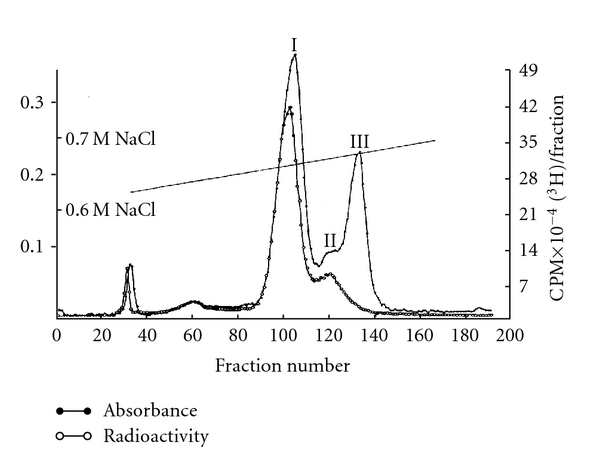
DEAE-Sephadex column chromatography of 4.5S RNA [38]. The 4.5S RNA from preparative gel electrophoresis was collected and chromatographed on a DEAE-Sephadex A-50 column. It was separated into 3 peaks but no radioactivity in 4.5S RNA III was detected, indicating the absence of accessible 2′-OH and 3′-OH in this molecule. The 4.5S RNA II may be the U6 RNA.

The labeled 4–8S RNAs were separated by preparative polyacrylamide gel electrophoresis (Figure 13) and DEAE-Sephadex column chromatography (Figure 14) to purify individual snRNAs (U1, U2, U3, 4.5S RNA I, II, and III, 5S RNA I, II, and III).

Alkaline hydrolysis of these RNAs produced 3′ end nucleoside trialcohol derivatives (Table 9) which were subsequently identified by paper chromatography. 

**Figure 15:**
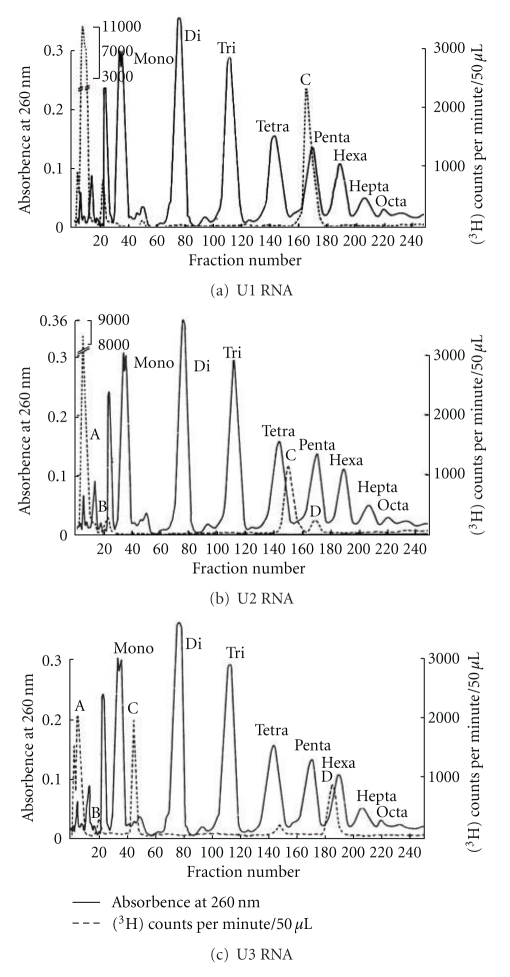
DEAE-Sephadex chromatography of 5′ oligonucleotide [37]. The U1 RNA, U2 RNA, and U3 RNA collected from preparative gel electrophoresis (Figure 13) were digested with RNase A and subjected to DEAE-Sephadex A-25 column chromatography. The radioactive peaks at nucleoside region were coming from 3′ ends and the radioactivities at the regions of penta-, tetra-, and hexanucleotides were from 5′ end labeling. These fragments were treated with T1 RNase and found to be shortened by one nucleotide only in U3 5′ oligonucleotide indicating that G was next to the terminal pyrimidine nucleotide.

The RNA that appeared to be pure for sequencing was 4.5S RNA I which had 87.4% U at the 3′ terminus and only 6.5% unknown radioactivity at the origin. Unexpectedly, U1, U2, U3, 4.5S RNA II, and some of 5S RNA (5S RNA III/U5) had ~50% labeling in alkaline-resistant fragments that did not move as nucleoside derivatives. The 4.5S RNA III was not labeled by this procedure suggesting a blocked 3′ end (Figure 14). The U1, U2, and U3 RNAs were labeled with tritium, digested with RNase A, and separated on a DEAE-Sephadex column (Figure 15). 

The oligonucleotides were digested with T_1_ RNase and rechromatographed, and only the U3 oligonucleotide was shortened by one nucleotide, indicating the presence of one G adjacent to RNase A susceptible pyrimidine [80]. In the course of sequencing U1, U2, U3 RNAs, it was found that the oligonucleotides with m_3_^2,2,7^G was coming from the 5′ end segments. The only way 2′3′ hydroxyls could be at 5′ end was 5′-5′ pyrophosphate linkage to the rest of the RNA molecules [36]. The RNase A and T_1 _RNase resistant oligonucleotides were digested with various enzyme combinations including snake venom phosphodiesterase, alkaline phosphatase, P_1_ RNase, T_2_ RNase, and U_2_ RNase into nucleosides. The component nucleosides were identified by mass spectrometry, U.V. spectroscopy, HPLC (high pressure liquid chromatography), paper chromatography, and thin layer chromatography. [12, 16, 37, 58].

### 7.2. Tritium Labeling of Nucleosides

The purified RNAs were digested with RNase A, snake venom phosphodiesterase, and alkaline phosphatase at pH 8.0, 37°C for 6 hours into nucleosides. The digest was treated with a 2X molar excess of NaIO_4_ and labeled with [^3^H]-KBH_4_ at pH 6 for 2 hours in the dark to produce trialcohol derivatives of nucleosides. All nucleosides with base modifications, except 2′-O-ribose modified, were labeled with tritium. The tritium-labeled trialcohol derivatives were separated by two-dimensional TLC (thin layer chromatography) on cellulose thin layers (Figure 5) [81]. The first dimension used a solvent of acetonitrile, ethylacetate, n-butanol, isopropanol, 6 N aqueous ammonia (7 : 2 : 1 : 1 : 2.7); the second dimension used a solvent of tert-amyl alcohol, methylethylketone, acetonitrile, ethylacetate, water, formic acid (sp.gr. 1.2) (4 : 2 : 1.5 : 2 : 1.5 : 0.18) [81, 82].

### 7.3. [^32^P] Labeling of RNA

**Figure 16:**
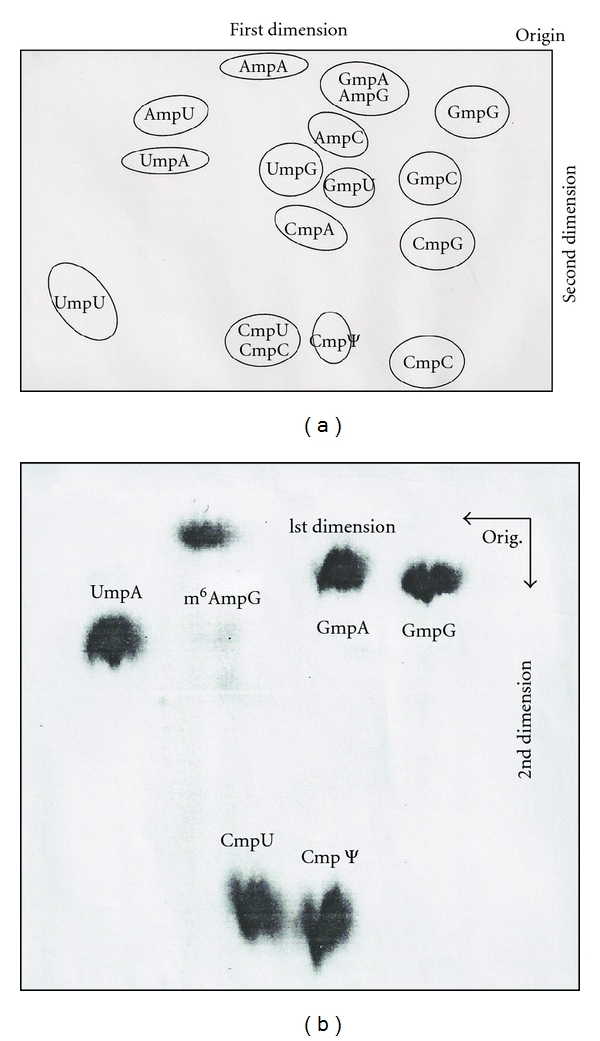
Two-dimensional separation of alkali stable dinucleoside monophosphate [18, 39]. (a) Standard dinucleoside monophosphates (NmpN) were separated on Whatman no.1 paper with the solvent systems ethylacetate-1-propanol-water (4 : 1 : 2, v/v) in the first dimension and in the second dimension with 2-propanol-water-concentrated ammonium hydroxide (7 : 2 : 1, V/V). (b) Autoradiograph of two-dimensional separation of alkali-stable dinucleoside monophosphate of U2 RNA from Novikoff hepatoma cell nuclei. The [^32^P]-labeled U2 RNA was hydrolyzed by 0.3 N NaOH, and the sample was separated on a DEAE-Sephadex column A-25 at pH 7.6. The dinucleotides were collected and treated with alkaline phosphatase. The alkali stable dinucleoside monophosphates were separated on Whatman no. 1 paper and autoradiographed with X-ray film.

The Novikoff hepatoma cells were transplanted intraperitoneally into male albino rats of the Holtzman strain weighing 200–250 g, obtained from Cheek Jones Company (Houston, Tex). After 5-6 days, the cells were harvested and washed with NKM solution (0.13 M NaCl, 0.005 M KCl, and 0.008 M MgCl_2_). Twenty milliliter (packed volume) of cells was incubated with 500 mCi of [^32^P]-orthophosphate in 1 liter of medium (phosphate free modified Eagle's minimal essential medium) for 9–16 hours [83]. Nuclear RNA was purified by sucrose gradient centrifugation, gel electrophoresis, and column chromatography [38]. The purified RNA was hydrolyzed with 0.3 N KOH, and alkaline-resistant oligonucleotides were separated on DEAE-Sephadex. The alkaline resistant dinucleotides were collected, treated with alkaline phosphatase, and identified by two-dimensional chromatography (Figure 16).

 The summary of modified nucleotides is in Table 1 [84].

## 8. Structural Determination of 5′ Oligonucleotides

**Figure 17:**
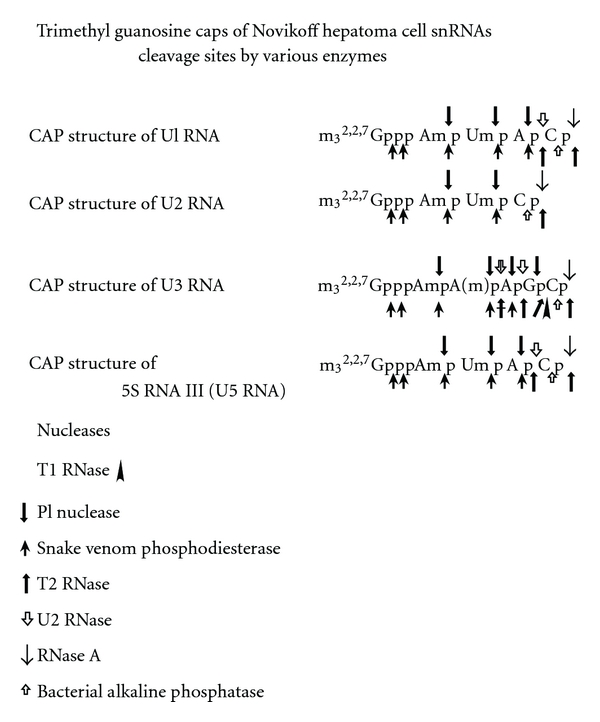
Characterization of m_3_^2,2,7^G caps of snRNAs U1, U2, U3, and U5. The enzyme susceptible bonds are indicated with arrows. The split arrows indicate that some bonds without 2′-O-methylation can be cleaved but the ones with 2′-O-methylated ribose are not.

The structures of the 5′ ends of U1 RNA, U2 RNA, U3 RNA, and 5S RNA III (U5) are determined by the characteristics of chemical reactions and enzymatic susceptibilities (Figure 17). 

### 8.1. U1 RNA 5′ End Oligonucleotide

The U1 RNA labeled with [^3^H] by NaIO_4_ and [^3^H]-KBH_4_, digested with RNase A, showed enzyme-resistant oligonucleotide eluting close to the pentanucleotide region on a DEAE column (Figure 15). The 5′ oligonucleotide was analyzed by UV, [^3^H], and [^32^P] methods. 

#### 8.1.1. The UV Analysis

**Figure 18:**
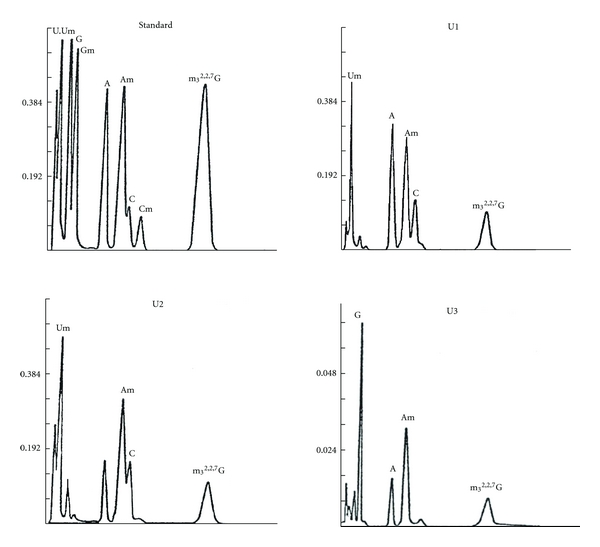
HPLC (High Pressure Liquid Chromatography) pattern of nucleosides of 5′ oligonucleotides of U1 RNA, U2 RNA, and U3 RNA [37]. The 5′ oligonucleotides of U1, U2, and U3 RNAs were obtained by digestion of T_1_ RNase and RNase A. The fragments were digested with snake venom phosphodiesterase and alkaline phosphatase. The nucleosides produced were separated by high pressure liquid chromatography (Varian Aerograph Liquid Chromatograph LCS-1000) at 55°C, 700–800 p.s.i. with 0.4 M ammonium formate (pH 3.5). Absorbance at 254 nm was recorded.

The 5′ oligonucleotides from U1 RNA, obtained by RNase A and RNase T1, were digested with snake venom phosphodiesterase and alkaline phosphatase. The nucleosides produced were separated on HPLC (high pressure liquid chromatography) [strongly basic cation exchange (quaternary amine)]. As shown in Figure 18, the amount of nucleoside ratio was 1.0, 1.2, 1.2, 0.7, and 0.9 for Am, A, Um, m_3_^2,2,7^G, and C, respectively, for U1 5′ oligonucleotide.

#### 8.1.2. The [^3^H] Method

**Figure 19:**
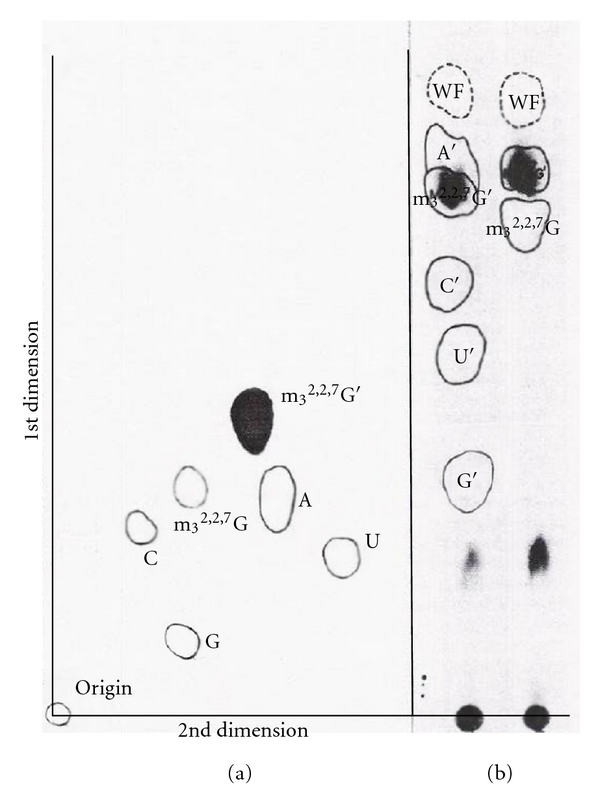
Identification of m_3_^2,2,7^G′ [16]. (a) Fluorogram of two-dimensional thin layer chromatography of [^3^H]-labeled nucleoside trialcohol derivative (N′) released from U1 RNA 5′ fragment. It was identified as m_3_^2,2,7^G′ with standard. (b) Fluorogram of chromatographic separation of [^3^H]-labeled nucleoside trialcohol derivative on Whatman 3MM paper. The [^3^H]-labeled compound was identified as m_3_^2,2,7^G′ with standard.

The [^3^H]-labeled U1 RNA 5′ oligonucleotide, following digestion with snake venom phosphodiesterase and alkaline phosphatase, was separated by chromatographic methods with standards. Two-dimensional TLC (thin layer chromatography) and paper chromatography demonstrated that the [^3^H] labeled compound is a trimethylguanosine derivative (Figure 19).

#### 8.1.3. ^32^P-Labeled 5′ Oligonucleotide from U1 RNA

**Figure 20:**
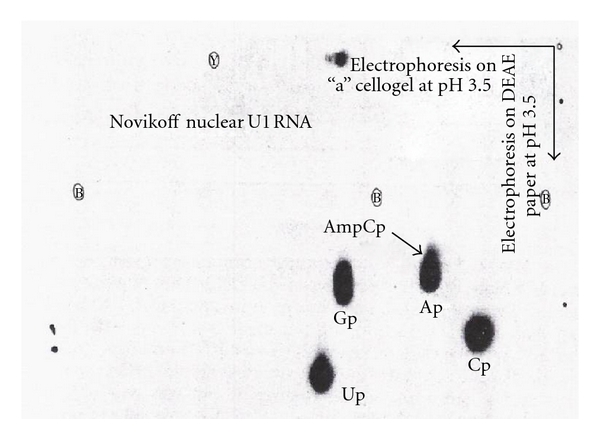
2D map of U1 RNA digested with T_2_ RNase and U_2_ RNase [16]. The U1 RNA uniformly labeled with [^32^P] was digested with T_2_ RNase and U_2_ RNase. The resistant 5′ fragment (spot “a”) was separated from the rest of the hydrolysate by two-dimensional electrophoresis. The first dimension was on cellogel at pH 3.5, and the second dimension was on DEAE paper in 5% acetic acid-NH_4_ acetate at pH 3.5.

The ^32^P-labeled RNA was digested with T_2_ and U_2_ RNase, and digestion products were separated by two-dimensional electrophoresis. The first dimension was on cellogel at pH 3.5, and the second dimension was on DEAE paper at pH 3.5 (Figure 20). 

**Figure 21:**
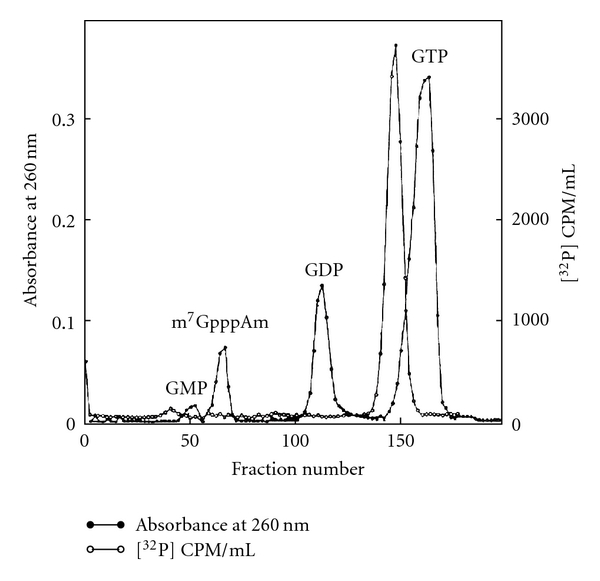
DEAE column chromatography of U1 5′ oligonucleotide [16]. Spot “a” in Figure 20 was eluted and digested with alkaline phosphatase. The product was chromatographed on DEAE-Sephadex A-25 with GMP, GDP, and GTP. The fragment chromatographed at GTP region.

Spot “a” was eluted and treated with alkaline phosphatase and chromatographed with GMP, GDP, and GTP standards. The ^32^P-labeled 5′ oligonucleotide was chromatographed in the GTP region on a DEAE-Sephadex column (Figure 21).

**Figure 22:**
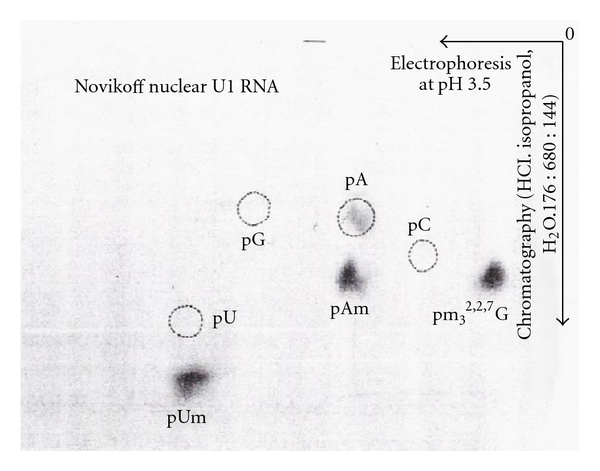
Nucleotide composition of U1 5′ oligonucleotide [16]. The peak from the GTP region was digested with snake venom phosphodiesterase and separated on Whatman 3MM paper by electrophoresis at pH 3.5 (5% acetic acid adjusted pH to 3.5 with ammonium hydroxide) and chromatography in the second dimension with a solvent system consisting of isopropyl alcohol, HCl, and H_2_O in the ratio of 680 : 176 : 144 by volume. Autoradiography was performed using X-ray film.

The oligonucleotide peak from the GTP region was digested with snake venom phosphodiesterase and separated by electrophoresis in the first dimension followed by chromatography on second dimension (Figure 22).

**Figure 23:**
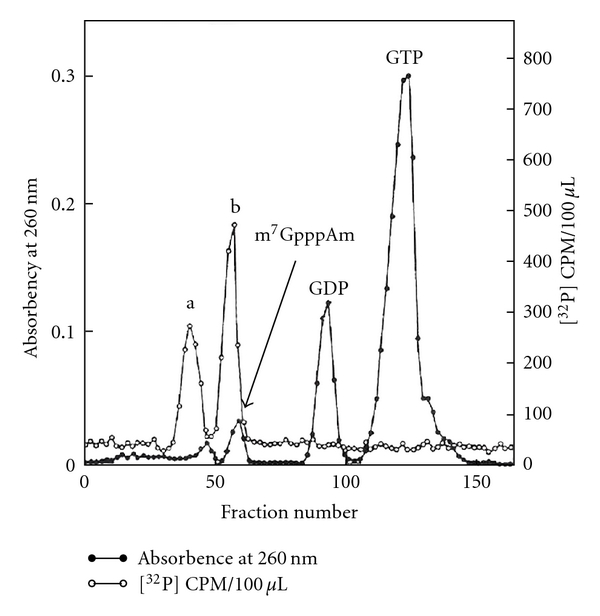
Cap core chromatography [16]. The 5′ oligonucleotide eluted from the GTP region (Figure 21) was digested with P1 RNase and chromatographed on a DEAE-Sephadex A-25 column. Two peaks “a” (mononucleotides pUm and pA) and “b” (cap core m_3_^2,2,7^GpppAm) were observed.

The ^32^P activity ratio was 1.00, 1.11, 1.25, 0.53, and 1.14 for pm_3_^2,2,7^G, pAm, pUm, pA, and Pi, respectively. The peak from the GTP region in Figure 21 digested with RNase P1 produced pUm, pA (peak a in Figure 23), and cap core m_3_^2,2,7^GpppAm (peak b in Figure 23).  Table 10 shows the radioactivity distribution in peaks a and b in Figure 23.

**Figure 24:**
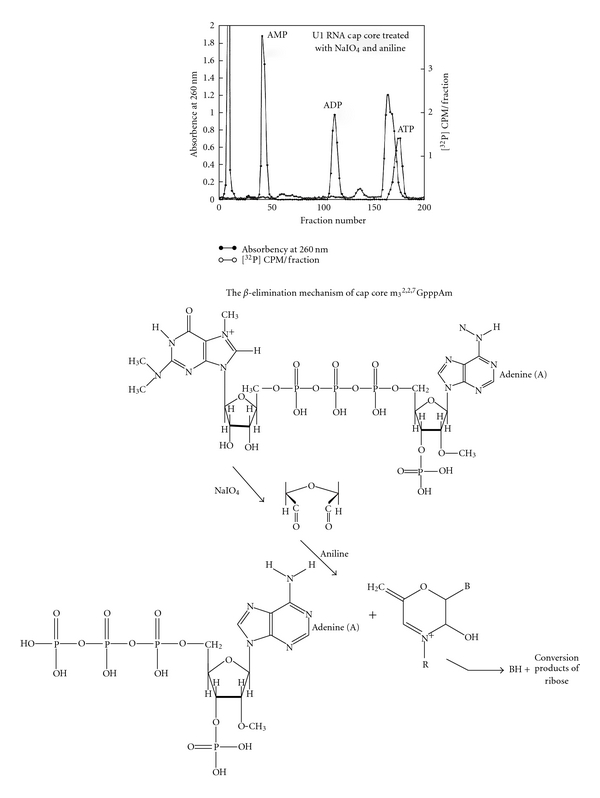
The *β*-elimination of cap core [16]. The cap core (peak b from Figure 23) was treated with NaIO_4_ and aniline to remove m_3_^2,2,7^G by *β*-elimination reaction. The remaining nucleotide was chromatographed in the ATP region indicating it is pppAm. This proved that the cap core was m_3_^2,2,7^GpppAm.

For the analysis of a number of phosphates in cap core (peak b), the cap core was treated with NaIO_4_ and aniline to remove m_3_^2,2,7^G by *β*-elimination reaction (Figure 24). 

The product was chromatographed on a DEAE column with standard AMP, ADP, and ATP. The product was eluted close to ATP, indicating that it is pppAm. This experiment proved that the 5′ oligonucleotide structure is **m**_3_^2,2,7^ **GpppAmpUmpApCp**. 

### 8.2. U2 RNA 5′ End Oligonucleotide

The U2 RNA labeled with NaIO_4_ and [^3^H]-KBH_4_ was digested with RNase A. The labeled oligonucleotide eluted around the tetranucleotide region (Figure 15). The 5′ oligonucleotide was analyzed by UV, [^3^H], and [^32^P] methods. 

#### 8.2.1. UV Analysis

**Figure 25:**
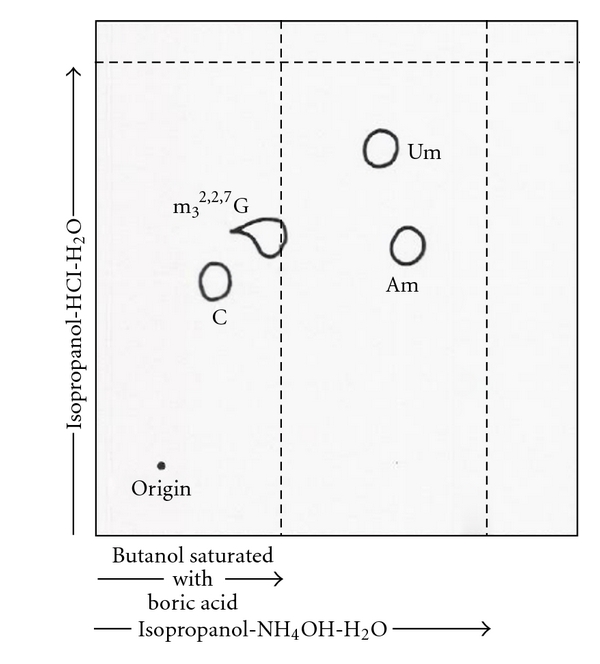
Two-dimensional chromatography in borate system [12]. The nucleoside mixture from U2 RNA 5′ oligonucleotide (RNase A product) was obtained by digestion with snake venom phosphodiesterase and alkaline phosphatase. The resulting nucleosides were separated with the borate system. Um and Am migrated through the butanol-boric acid while the m_3_^2,2,7^G and C, which form borate complexes, were retained in the butanol-boric acid phase.

The 5′ oligonucleotide obtained by complete RNase A digestion was analyzed for its base composition. The purified 5′ oligonucleotide was digested with snake venom phosphodiesterase followed by alkaline phosphatase. The digestion product (nucleosides) was separated by HPLC. The composition was Am, Um, C, and m_3_^2,2,7^G in a ratio of 1.0, 1.3, 1.1, and 0.96, respectively, (Figure 18) [12]. These nucleosides were also separated by two-dimensional TLC in a borate system. Um and Am migrated through the butanol-boric acid while the m_3_^2,2,7^G and C, which form complexes with borate, were retarded in the butanol-boric acid phase (Figure 25). 

The UV spectra of pm_3_^2,2,7^G were typical of a trimethyl G nucleotide (Figure 8). The mass spectrometry of the unknown nucleoside from U2 RNA 5′ fragment was identified as m_3_^2,2,7^ trimethylguanosine (Figure 9).

#### 8.2.2. [^3^H]-Labeled U2 RNA 5′ Oligonucleotide

**Figure 26:**
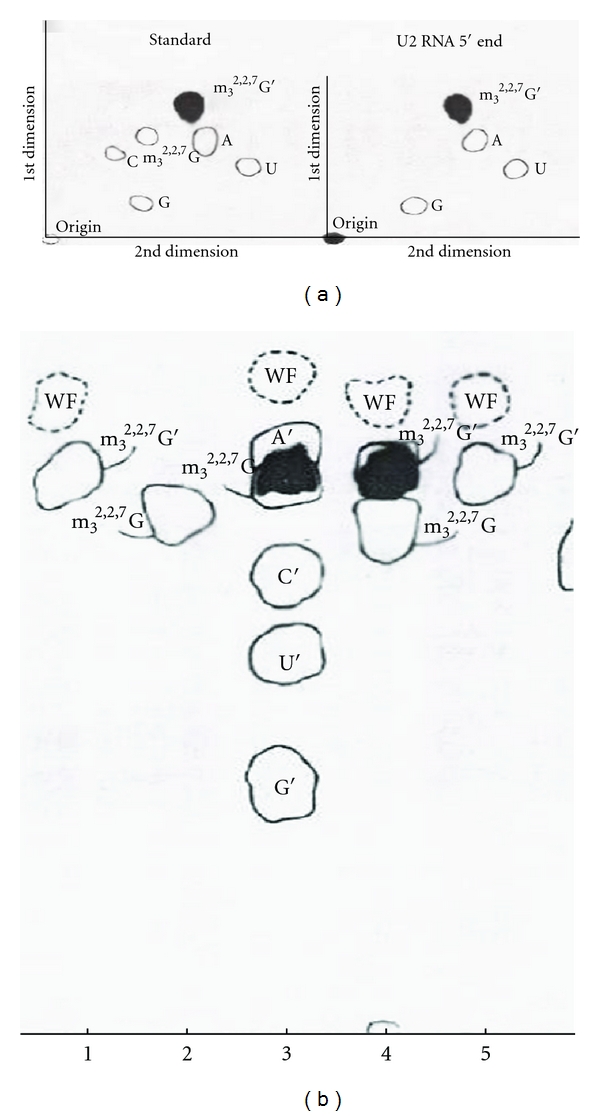
Fluorograph of [^3^H]-labeled trialcohol derivatives of m_3_^2,2,7^G from U2 RNA 5′ end [12]. (a) Two-dimensional thin layer chromatography. (b) One-dimensional paper chromatography. In both systems, the labeled compound was m_3_^2,2,7^G′. (See text).

The purified U2 RNA, labeled with NaIO_4_ and [^3^H]-KBH_4_ methods, was digested with RNase A and 5′ oligonucleotide purified by DEAE-Sephadex column chromatography (Figure 15). The purified 5′ oligonucleotide was digested with snake venom phosphodiesterase followed by alkaline phosphatase. The nucleosides obtained were separated on two-dimensional TLC [12] and 3MM paper chromatography. The tritium-labeled compound was identified as a trialcohol derivative of m_3_^2,2,7^G (Figure 26).

#### 8.2.3. [^32^P]-Labeled U2 RNA 5′ Oligonucleotide

**Figure 27:**
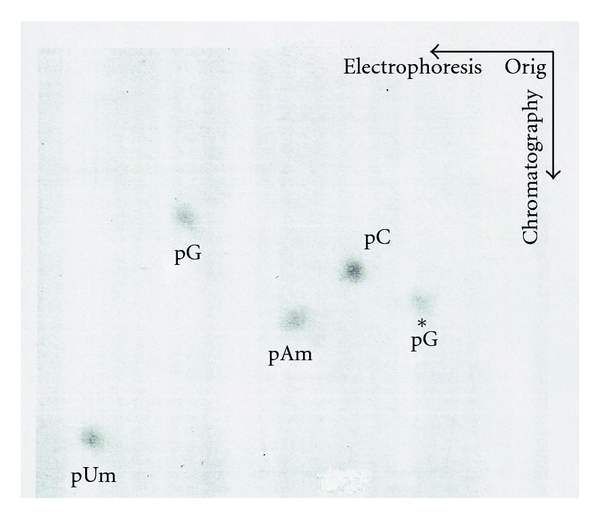
Autoradiograph of nucleotides from [^32^P]-labeled U2 RNA 5′ fragment [12]. The [^32^P]-labeled U2 RNA 5′ fragment (T1 RNase digestion) was treated first with alkaline phosphatase and then with snake venom phosphodiesterase. This mixture of mononucleotide products was separated by electrophoresis followed by chromatography. Approximately equal amounts of pm_3_^2,2,7^G, pAm, pUm, pC, and pG were observed (Table 11).

The [^32^P]-labeled U2 RNA was digested with T_1_ RNase or RNase A. Half of each 5′ oligonucleotide was digested with alkaline phosphatase. Oligonucleotides were subsequently digested with snake venom phosphodiesterase, and the resulting 5′ nucleotides were separated first by electrophoresis and second by chromatography (Figure 27). The ratio of [^32^P] counts is shown in Table 11. 

**Figure 28:**
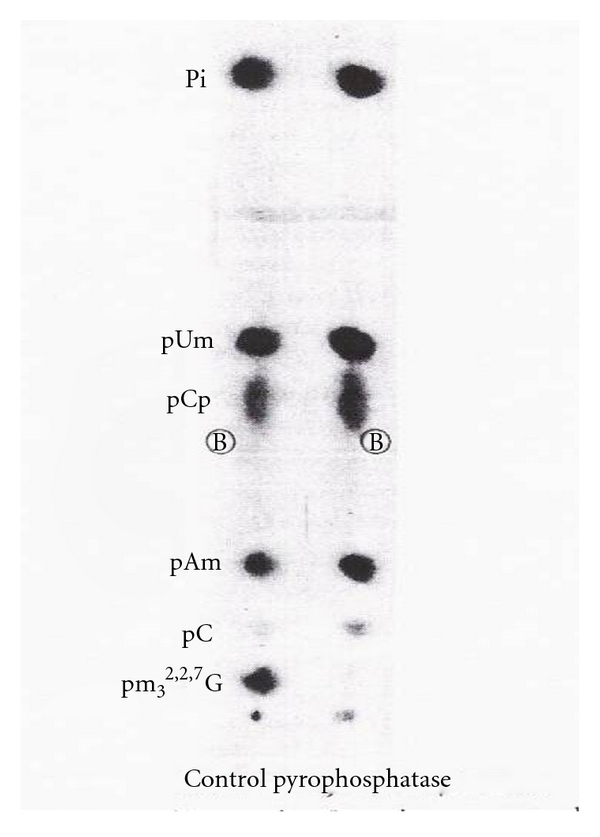
The susceptibility of 5′ cap to pyrophosphatase [12]. The [^32^P]-labeled 5′ oligonucleotide obtained from U2 RNA by RNase A was digested with pyrophosphatase and base composition was analyzed by snake venom phosphodiesterase digestion. This digestion released m_3_^2,2,7^G from the 5′ fragment indicating that m_3_^2,2,7^G is linked by a pyrophosphate linkage.

The U2 RNA 5′ oligonucleotide obtained by RNase A was subjected to digestion with pyrophosphatase (*Crotalus adamanteus* venom type II, Sigma). The remaining oligonucleotide did not have m_3_^2,2,7^G, indicating that the m_3_^2,2,7^G is linked by pyrophosphate linkage (Figure 28).

From these data the 5′ end oligonucleotide from U2 RNA has been deduced to be **m**_3_^2,2,7^ **GpppAmpUmpCpGp**. 

### 8.3. U3 RNA 5′ End Oligonucleotide

**Figure 29:**
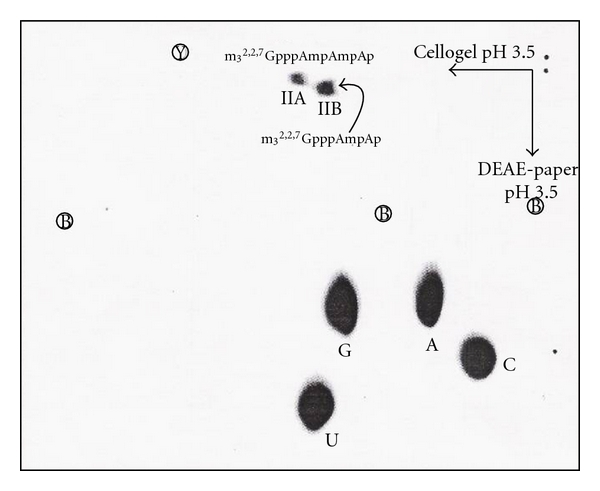
2D map of U3 RNA digest [58]. The [^32^P]-labeled U3 RNA was digested with T_2_ RNase and U_2_ RNase. It produced two 5′ fragments “11A” and “11B”.

The [^3^H]-labeled U3 RNA was digested with RNase A and or T_1_ RNase. The [^3^H]-labeled 5′ oligonucleotide obtained by RNase A digestion was eluted in the hexanucleotide region (Figure 15). The [^32^P]-labeled U3 RNA digested with T_2_ and U_2_ RNA produced 2 spots that were separated by two-dimensional electrophoresis (Figure 29). 

#### 8.3.1. UV Analysis

The 5′ oligonucleotide obtained from U3 RNA by digestion with RNase A and T_1_ RNase was isolated by column chromatography. The purified 5′ oligonucleotide was digested with snake venom phosphodiesterase and alkaline phosphatase. The nucleosides obtained were subjected to HPLC. The molar ratios of m_3_^2,2,7^G, Am, A, and G were 1.0, 1.7, 1.1, and 1.0, respectively (Figure 18).

#### 8.3.2. [^3^H] Analysis

**Figure 30:**
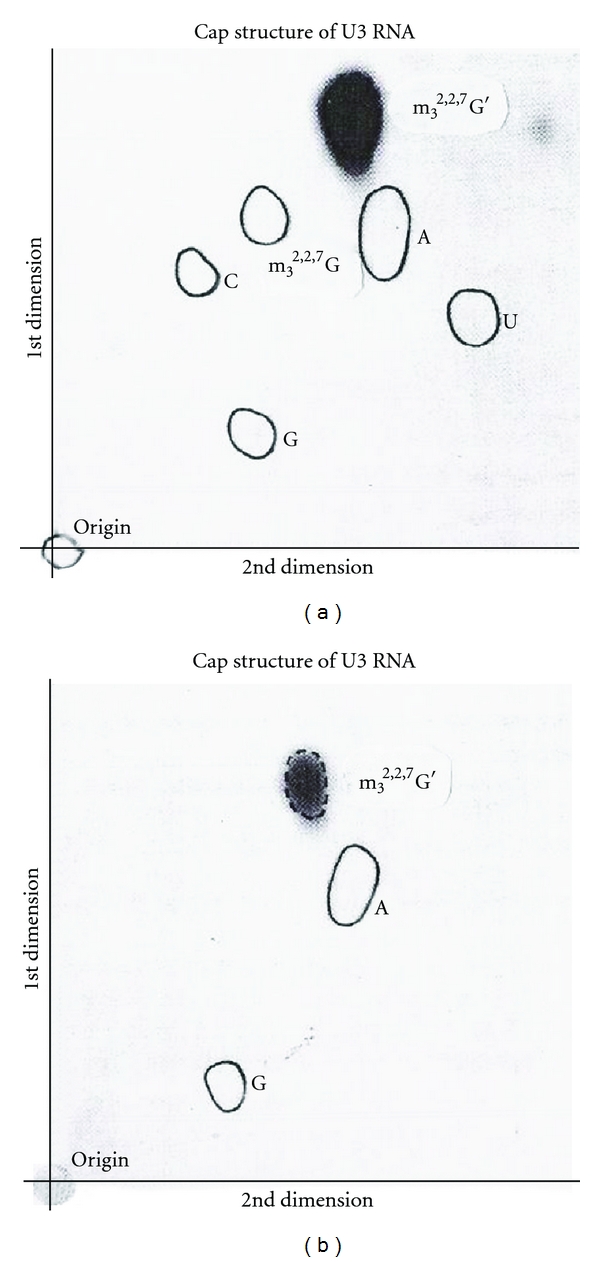
m_3_^2,2,7^G′ identification from U3 RNA 5′ end fragment [58]. The [^3^H]-labeled U3 RNA 5′ fragment obtained by RNase T_1_ and RNase A was digested with snake venom phosphodiesterase and alkaline phosphatase. The nucleoside mixture was separated by two-dimensional thin layer chromatography with standard nucleoside mixture in (a) and with the trialcohol derivative of m_3_^2,2,7^G′ in (b). The released nucleoside trialcohol derivative was identified as m_3_^2,2,7^G′ by fluorography.

The intact U3 RNA, labeled with NaIO_4_ and [^3^H]-KBH_4_ methods, was digested with RNase A and chromatographed on DEAE-Sephadex (Figure 15). Subsequent digestion by T_1_ RNase released only one nucleotide from the RNase A oligonucleotide, indicating that the G was adjacent to a RNase A susceptible pyrimidine. The purified 5′ oligonucleotide obtained after T_1_ RNase and RNase A was digested with snake venom phosphodiesterase followed by alkaline phosphatase. The nucleosides and trialcohol derivatives were separated by TLC (Figure 30). The trialcohol derivative of m_3_^2,2,7^G indicates that this nucleotide has free 2′ and 3′ OH at the end of the intact molecule.

#### 8.3.3. [^32^P] Analysis

**Figure 31:**
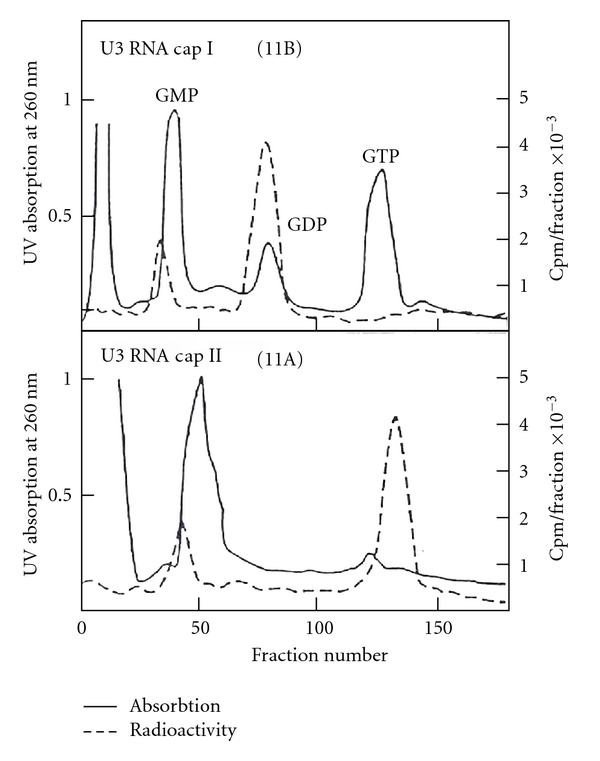
U3 RNA cap I and cap II chromatography [58]. The 5′ fragment from Figure 29 was eluted and treated with alkaline phosphatase and chromatographed on DEAE-Sephadex A-25 with standards of GMP, GDP, and GTP. Component “11B” was eluted in the GDP (cap I, m_3_^2,2,7^GpppAmA) region and “11A” (cap II, m_3_^2,2,7^GpppAmAmA) was eluted in the GTP region.

The [^32^P]-labeled U3 RNA digested by T_1_ RNase and U_2_ RNase was separated by two-dimensional electrophoresis (Figure 29). The enzyme-resistant oligonucleotides 11A and 11B were eluted from the paper and treated with alkaline phosphatase. The products were chromatographed on DEAE-Sephadex A-25 with GMP, GDP, and GTP markers. The 11A (cap II) was eluted at GTP region and 11B (cap I) was eluted at GDP region, indicating that 11B has one nucleotide less than 11A (Figure 31). 

From these data, obtained by UV, [^3^H], and [^32^P] experiments, the U3 RNA 5′ oligonucleotide sequence has been deduced to be **m**_3_^2,2,7^ **GpppAmpA(m)pApGpCp**. 

### 8.4. 5S RNA III (U5 RNA) 5′ End Oligonucleotide

The oligonucleotide sequence was deduced as in the case of U1 RNA. The structure is identical to the U1 5′ oligonucleotide **m**_3_^2,2,7^ **GpppAmpUmpApCp**. 

## 9. RNA Signature Modifications for Different RNA Classes

### 9.1. End Modifications

#### 9.1.1. 5′ End

**(a) According to Chemical Nature of Caps**

5′ Trimethylguanosine cap for the snRNA,
5′ 7-mehtylguanosine cap for the mRNA,
5′ 2,7 dimethylguanosine cap of virus and nematode RNAs
5′ mpppG of U6 RNA.

**(b) According to Flanking Nucleotide Modification of Caps.**

(See Table 13).

**(c) 5′ End Uncapped RNA**

(pppNp) for primary transcripts such as 4.5S RNA I, 5S RNA, and Alu RNA. (pNp) 5′ end for processed RNAs such as Alu RNA, 5S RNA, tRNA, YRNA.

#### 9.1.2. 3′ End

3′2′-O-methylated; 4.5S RNA III
3′ poly-A; mRNA, lncRNA
3′ poly-U; polymerase III transcripts such as 4.5S RNA I, 5S RNA, and others
3′ CCA; tRNA, U2 RNA.

### 9.2. Internal Modifications

The most colorful modifications are in tRNAs that contain methyl, formyl, acetyl, isopentyl, threonyl, carbamoyl, and other groups and modifications by pseudouridylation, deamination, reduction, or thiolation. Focusing on recent findings for snRNA, m_3_^2,2,7^G capping reactions are very interesting because trimethylguanosine is found only in noncoding RNA cap structures, although some nematode mRNA species also contain m_3_^2,2,7^G caps. The snRNAs are less abundant (10^5^ copies) than ribosomal RNA or tRNA (10^6^ copies). Isolating large amount of RNA can be a hurdle to overcome. Massive preparative procedures and syntheses were pivotal for the thorough analysis of these modifications. The 2′-O-modifications occur mostly internally, and 3′ Um was also found in 4.5S RNA III. The RNA ribose with 2′-O-methylation confers resistance to enzymatic digestion by such enzymes as RNase A, RNase T_1_, RNase U_2_, and RNase T_2 _. They are also resistant to alkaline hydrolysis, and the alkaline hydrolysates can be separated into di-, tri-, and tetranucleotides by column chromatography and then by two-dimensional paper chromatography (Figure 16). Other enzymes which can cleave 2′-O-methylated nucleotides are snake venom phosphodiesterase, P1 nuclease, and spleen phosphodiesterase. These are valuable tools for sequencing. 

## 10. Presence of m_3_^2,2,7^G Caps in RNA Species

### 10.1. Nucleolar RNA

Initially, the m_3_^2,2,7^G cap containing snoRNA was found in U3 RNA [36]. Since then C/D snoRNA and H/ACA snoRNA have been discovered exponentially. The snoRNAs are transcribed from monocistronic as well as polycistronic independent positions as well as intronic regions of mRNA, especially the genes coding ribosomal proteins. In vertebrates, there have been >76 snoRNAs that have been reported, but only U3, U8, and U13 snoRNAs have been reported to have m_3_^2,2,7^G caps [33, 88]. In yeast, there are at least 17 m_3_^2,2,7^G cap containing snoRNAs out of more than 76 snoRNAs. It was also reported that some snoRNA precursors, such as pre-snoRNAs 50, 64, and 69, have the m_3_^2,2,7^G cap, but mature snoRNA 50, 64, and 69 do not have m_3_^2,2,7^G caps. The maturation process cleaves the 5′ fragment by Rnt1 (RNase III like enzyme), and trimming is performed by 5′ → 3′ exonuclease Xrn1 and Rat 1 [89].

### 10.2. Spliceosomal snRNAs

These include U1, U2, U4, U5, and U6 snRNAs. All of these except U6 contain the m_3_^2,2,7^G cap, and U6 has the mpppG cap instead. They are present in complexes as RNP with proteins specific for each RNA as well as some common snRNP proteins such as the Sm proteins. Functionally, U1 RNP acts at 5′ splice sites and U2 RNA at branch sites including 3′ splice sites. U4, U5, and U6 snRNAs enter the spliceosomal intermediate as a tri-snRNP complex.

### 10.3. Human Telomerase RNA (hTR)

Human telomerase RNA has a structure containing the H/ACA motif with 8 conserved regions (CR 1–8) [92]. The CR7 contains the CAB box (Cajal body box) consensus sequence of UGAG and directs the RNA localization into the CB (Cajal body). The Tgs1 (trimethyl guanosine synthase) is also present in the Cajal body and may be responsible for the m_3_^2,2,7^G cap formation. Not all Cajal bodies contain the hTR, and it may be a transient localization for the maturation of hTR in the Cajal body. In the absence of Tgs1, the telomere of yeast *S. cerevisiae* has elongated single-stranded 3′ overhangs and TLC1 (1200 nt telomerase RNA) lacks the m_3_^2,2,7^G cap. The absence of Tgs1 causes premature aging of yeast [93, 94].

### 10.4. C. elegans SL RNA

**Figure 32:**
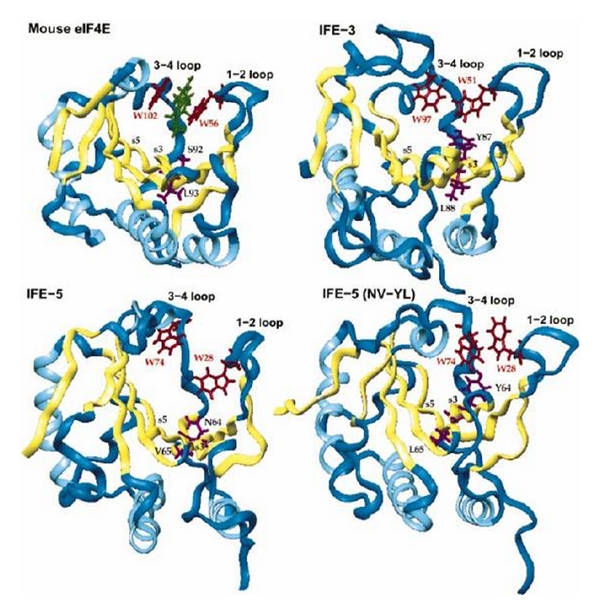
Molecular models of IFE-3, IFE-5, and IFE-5 (NV-YL) in comparison with mouse eIF4E [85]. The differences between the m^7^G cap binding pocket and m_3_^2,2,7^G cap binding pocket are illustrated by differences in the 3-4 loop configuration.

*C. elegans* has mRNA with the m^7^G cap as well as m_3_^2,2,7^G cap, and the expression is regulated differentially. The genes for protein coding are monocistronic as well as polycistronic, and introns are much smaller than observed in mammalian cells. The polycistronic genes contain 2–8 operonic genes regulated by the same promoters. Some gene products are not processed, and others are spliced by cis-splicing as well as transsplicing. The transsplicings are carried out by SL RNA 1 or SL RNA 2. The approximately 110 SL RNA 1 genes are in tandem in chromosome V. The SL RNA 2 is derived from SL RNA 1 and there are ~18 dispersed genes with a variety of variant SL2 RNAs (some are called SL3, SL4, etc.). They are all 100–110 nucleotide long and contain m_3_^2,2,7^G caps and Sm protein binding sites. These pre-mRNAs, containing 5′ outron (monocistronic and 5′ first gene in polycistronic operonic genes), are transspliced by SL RNA 1 and internal operonic pre-mRNAs are mostly transspliced by SL RNA 2 and these genes have typically U-rich sequence containing ~100 bp spacers between two cleavage sites. The internal mRNA gene of polycistronic operonic genes, lacking a spacer, is transspliced always by SL RNA I [95, 96]. The transspliced mRNA contains a m_3_^2,2,7^G cap containing 22 nucleotides of SL RNA at their 5′ ends. The SL RNA (splice leader RNA) has a m_3_^2,2,7^G cap and Sm protein binding sites. The nematode *C. elegans* has 5 eIF4E isoforms of cap binding proteins. They are IFE-1 (m^7^G cap and m_3_^2,2,7^G cap binding), IFE-2 (m^7^G cap binding, but competed by the m_3_^2,2,7^G cap), IFE-3 (m^7^G cap binding only), IFE-4 (m^7^G cap binding only), and IFE-5 (m^7^G cap and m_3_^2,2,7^G cap binding). The homolog amino acids W56 and W102 stacking the m^7^G caps in mice eIF4E are W51 and W97 in IFE-3 and W28 and W74 in IFE-5 (Figure 32).

The differences in 3-4 loop configuration between IFE-5 and IFE-3 are N64Y/V65L. The changes in IFE-5 amino acid asparagine 64 to tyrosine and valine 65 to leucine change binding properties more to m^7^G cap binding than to m_3_^2,2,7^G cap binding. IFE-5 has 4 cysteines, and its conformation is governed by disulfide bond formation. It is suggested that the cap binding cavity is altered to produce a smaller cavity that discriminates against the m_3_^2,2,7^G cap binding [85]. These may provide translational regulation of m7G cap mRNA and transspliced m_3_^2,2,7^G cap mRNA in *C. elegans*.

## 11. Synthesis of m_3_^2,2,7^G Cap

Trimethylguanosine cap synthesis is carried out by multiple steps involving modifications. Trimethyl G caps are present in snRNAs involved in splicing and also in snoRNA involved in rRNA processing and modifications such as Ψ formation (H/ACA snoRNA) or 2^**'**^-O-methylation (C/D snoRNA). These include U1, U2, U4, and U5 spliceosomal RNAs, and U3, U8, and U13 nucleolar RNAs. Recently, telomerase RNA (*S. cerevisiae* TLC1) has also been reported to have a trimethylguanosine cap structure. The trimethyl-G caps are formed on cap 0 or cap I of m^7^G caps of pre-snRNAs by dimethylation of N2 position by trimethylguanosine synthase (Tgs1). The Tgs1 has been found to be in the Cajal body and cytoplasm. The U3 snoRNA is hypermethylated in the Cajal body, and U1, U2, U4, and U5 snRNA have been reported to be hypermethylated in the cytoplasm. 

### 11.1. The m^7^G Cap Formation

The RNA polymerase initiates the RNA transcription with 5′ triphosphate nucleotides and in a majority with purine nucleotides of ATP or GTP. The capping reaction in a polymerase II system occurs cotranscriptionally within the nascent transcript of ~30–50 nucleotides. The guanylyltransferase is attached to heptad (YSPTSPS) repeats of CTD of RNA polymerase II. It was reported with cloned mouse guanylyltransferase and synthetic heptad repeats that the serine 5 phosphorylated 6 heptad repeats stimulated guanylyltransferase activity 4-fold. Serine 2 phosphorylation also binds the guanylyltransferase but did not stimulate enzyme activity [97]. The capping enzymes contain RNA triphosphatase and RNA guanylyltransferase in the same molecule, but methylating enzymes are in different protein and occurs in separate steps.

The enzymes involved are RNA triphophosphatase and RNA guanylyltransferase, which can be found in the same enzyme, catalyze removal of one phosphate from pppNp initiation nucleotide, and transfer GMP from GTP through intermediary GMP-lysine phosphamide enzyme complex. The RNA guanyl 7 methyltransferase methylates the guanine at N7 position. The RNA 2′-O-methyltransferase methylates penultimate nucleotide 2′ OH, producing the cap 1 structure. In rat liver, it has been reported that 2′-O-methylation may precede the guanosine N7 methylation [98]. 

The capping reactions by mammalian and shrimp capping complexes (HeLa cell, rat liver, calf thymus, and shrimp) [98] have been reported as below:

**RNA Triphosphatase and Guanylyltransferase**

The monomer of the 69–73 kDa protein has functions of RNA triphosphatase and RNA guanylyltransferase activity.

(1) $$\text{pppNpNpNpNp-}\overset{\text{RNA}\;\text{triphosphatase}}{\to }\text{ppNpNpNpNp-}$$

(2) $$\text{GTP}+\text{RNA}\;\text{guanylyltransferase}\overset{{\text{Mg}}^{++}}{\to }\text{GMP-}\left(\text{phosphamide}\right)\text{-E}+\text{ppi}$$

(3) $$\text{GMP-E}+\text{ppNpNpNpNp-}\overset{{\text{Mg}}^{++}}{\to }\text{GpppNpNpNpNp-}+\text{RNA}\;\text{guanylyltransferase}$$

(4) $$\text{GpppNpNpNpNp-}+\text{AdoMet}\overset{\text{RNA}\;2'\text{-O-methyltransferase}}{\to }\text{GpppNmpNpNpNp-}+\text{AdoHcy}$$

(5) $$\text{GpppNmpNpNpNp-}+\text{AdoMet}\overset{\text{RNA}\;\text{guanyl}\;7\text{-methyltransferase}}{\to }{\text{m}}^{7}\text{GpppNmpNpNpNp}+\text{AdoHcy}$$

Some of the capping enzymes (vesicular stomatitis virus, spring viremia of carp virus) use the substrate monophosphorylated 5′ end (pNpNpNpNp-) [99, 100], and 7-methylation occurs after the 2′-O-methylation has taken place.

From HeLa cells, two enzymes forming cap I from cap 0 and cap II from cap I have been purified and characterized [101].

### 11.2. Cap I Methyltransferase

This enzyme is present in both the nucleus (29.3 units/mg) and cytoplasm (3.74 units/mg) and cap II methyltransferase is exclusively in the cytoplasm (4.62 units/mg). Cap I methyltransferase uses GpppA(pA)_n_, m^7^GpppA(pA)_n_, m^7^GpppApGp, m^7^GpppApGpUp, and RNA with type 0 cap as substrates but not m^7^GpppA or GpppA. The substrate required for cap I formation should be at least a trinucleotide. 

The order of 7-methylation of ultimate G nucleotide and 2′-O-methylation of penultimate nucleotide is uncertain, and both pathways may occur.

### 11.3. Cap II Methyltransferase

**Figure 33:**
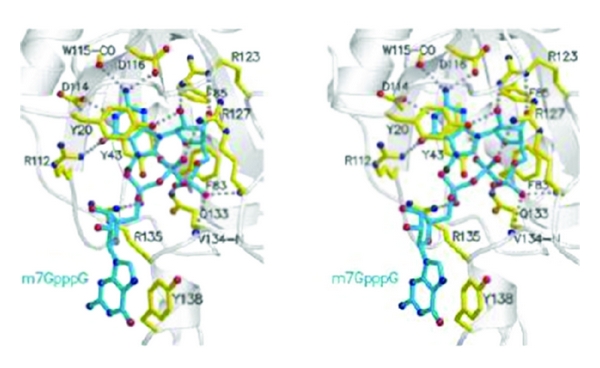
CBP20 (cap binding protein 20) binding to m^7^G cap [86, 87]. The m^7^G is stabilized by stacking energies between tyrosine 20 and tyrosine 43.

This enzyme is present only in the cytoplasm and converts cap I to cap II. The mature mRNA with 5′ m^7^G cap and 3′ polyadenylation is then transported into the cytoplasm as a complex with CBC20/80, PHAX, and Crm1-RanGTP. The m^7^G cap binds to CBC20 (156 amino acids) in complex with CBC80 (790 amino acids). The crystal structure of the CBC20/80 complex in association with m7G cap has been reported [86, 87]. The CBC20 is in an unfolded form in the absence of CBC80. The CBC80 has 3 domains, each containing consecutive 5-6 helical hairpins resembling the MIF4G (middle domain of eIF4G). The CBC20 has a typical RRM motif and binds between domains 2 and 3 of CBC80. The m^7^G cap is sandwiched between Tyr 43 and Tyr 20. And Phe 83, Phe 85, and Asp 116 have essential role for m^7^G cap binding. Asp 116 and Trp 115 interact with the N2 amino group and confer specificity of the m^7^G cap for other structures (Figure 33).

In the cytoplasm, the m^7^G cap plays a role in the initiation of translation by binding to eIF4E which complexes with eIF4A and eIF4G. The exact mechanism of exchange is not known but CBC80 has binding capacity for PHAX or eIF4G and dissociation of CBC80 from CBC20 makes CBC20 become disordered [86, 87].

### 11.4. Maturation of snRNAs

The snRNAs synthesized by RNA polymerase II with m^7^G cap structures are transported into the cytoplasm in complex with CBP20/80, PHAX (phosphorylated adaptor for RNA export), the CRM1 (export receptor, chromosome region maintenance 1) or exportin 1 and RanGTP (Ras-related nuclear antigen). The snRNPs in the cytoplasm are trimethylated and processed. The mature RNA is reimported into the nucleus in a complex with the trimethyl G cap-specific binding protein snurportin 1 and snRNA binding proteins of Sm RNP and SMN proteins.

Despite immunofluorescent staining of U1 and U2 RNA exclusively in the nucleus [102], biochemical analyses have demonstrated that trimethylation and maturation of some snRNA takes place in the cytoplasm. 

The U1 snRNA [103] and U2 snRNA [104] have been shown to be hypermethylated in the cytoplasm in a Sm protein binding dependent manner. The Xenopus laevis U1 RNA, with the m^7^G cap, has been shown to be hypermethylated in HeLa cell cytoplasmic extracts and Sm binding site in U1 RNA is required [103]. The Tgs1 has been shown to bind to Sm proteins of Sm B and Sm D. The Xenopus laevis U2 RNA with m^7^G cap has been shown to be hypermethylated into the m_3_^2,2,7^G cap structure in enucleated xenopus oocytes [104]. In yeast and human HeLa cells, the Tgs1 for U3 RNA is localized in the nucleolar body of the nucleolus and Cajal bodies, respectively [105]. In the absence of Tgs1 or inactive Tgs1 in yeast, m^7^G capped unprocessed U1 RNA is retained in the nucleolus and splicing becomes cold temperature sensitive. The same enzyme is responsible for the U3 nucleolar RNA hypermethylation [106]. The consensus between yeast and human cells is the presence of a nucleolar body in yeast and Cajal body in HeLa cell. The hypermethylation and processing during maturation take place in the nucleolar body in yeast and Cajal body in HeLa cells [105, 106]. The sequence element “UGAG” (also found in the U3 RNA B box) has been reported as a CAB box (Cajal-body-specific localization signal). U3 RNA trimethylation is somewhat different from other snRNAs. The U3 RNA, which does not have Sm protein binding sites, has been shown to require an intact 3′ terminal stem structure for trimethylguanosine cap formation [107].

In HeLa cells, transfected U3 RNA gene products are trimethylated and mature U3 RNA is localized in the nucleolus. Immature U3 RNA, with both m^7^G and 3′ extension of 10–15 nucleotides, is detected in Cajal bodies. The nucleolar localization requires the CAB box, hypermethylation to m_3_^2,2,7^G cap, and maturation of the 3′ end [105]. Unlike U1 RNA and U2 RNA, U3 RNA has been shown to be retained in the nuclear compartment and does not go into the cytoplasm for its trimethylation reaction [105, 106, 108].

## 12. The Tgs1 (Trimethylguanosine Synthase 1)

### 12.1. Human Tgs1

The Tgs1, trimethylguanosine synthase in human, protein is 110 kDa and 852 amino acids in chain length. The gene is located in chromosome 8q11. The mRNA is 3.2 kb in length and produces a 110 kDa protein and ~65–70 kDa protein that is proteasome processed. The long form is in the cytoplasm, and the short isoform has been reported to be localized in the Cajal body within the nucleus. The Tgs1 has S-AdoMet methyltransferase signature motifs of X, I, II (include post 1 motif), III, IV, V, and VI [70, 106, 109, 110]. 

The human Tgs1 motifs are the following.

motif X is (a.a.665)DREGWFSVTPEKIAEHI/FA(a.a.682),
motif I is (a.a.693)VVVDAFCGVGGN(a.a.704),
motif II is (a.a.714)RVIAIDIDPV/IKI(a.a.725) and post 1 motif is VIAID which is responsible for S-AdoMet binding to the enzyme,
motif III is (a.a.740)KIEFICGDFLLLAS(a.a.753),
motif IV is (a.a.758/759)VVFLSPPWGGPDYA(a.a.771/772),
motif V is (a.a.785/786)DGFEIFRLSK(a.a.794/795),
motif VI is (a.a.798/799)NNIVYFLPRNADI(a.a.810/811),

It was reported that trimethylation catalytic activity is located in the C-terminal region (amino acids 631–852) and this region contains the S-AdoMet-dependent methyltransferase motifs. The tryptophan in motif 4 is involved in *π* stacking with m^7^G guanosine of the substrate. The motif 1 and post 1 motif are reported to interact with S-AdoMet. [110]. The C-terminal domain is localized in the Cajal body and binds to C/D-snoRNA- and H/ACA-snoRNA-associated proteins such as fibrillarin, Nop56, as well as dyskerin [110].

The N-terminal portion of the molecule (amino acids 1–~477) has been reported to contain GXXGXXI, a K-homology domain for RNA binding, and a motif for SmB and SmD1 binding. The Tgs1 has also been shown to interact with PRIP (proliferator-activated receptor-interacting protein), and the N-terminal portion (amino acids 1–384) of Tgs1 has been shown to have stimulatory effects on transcription of PPAR*γ* and RXR*α* [109, 110].

**Figure 34:**
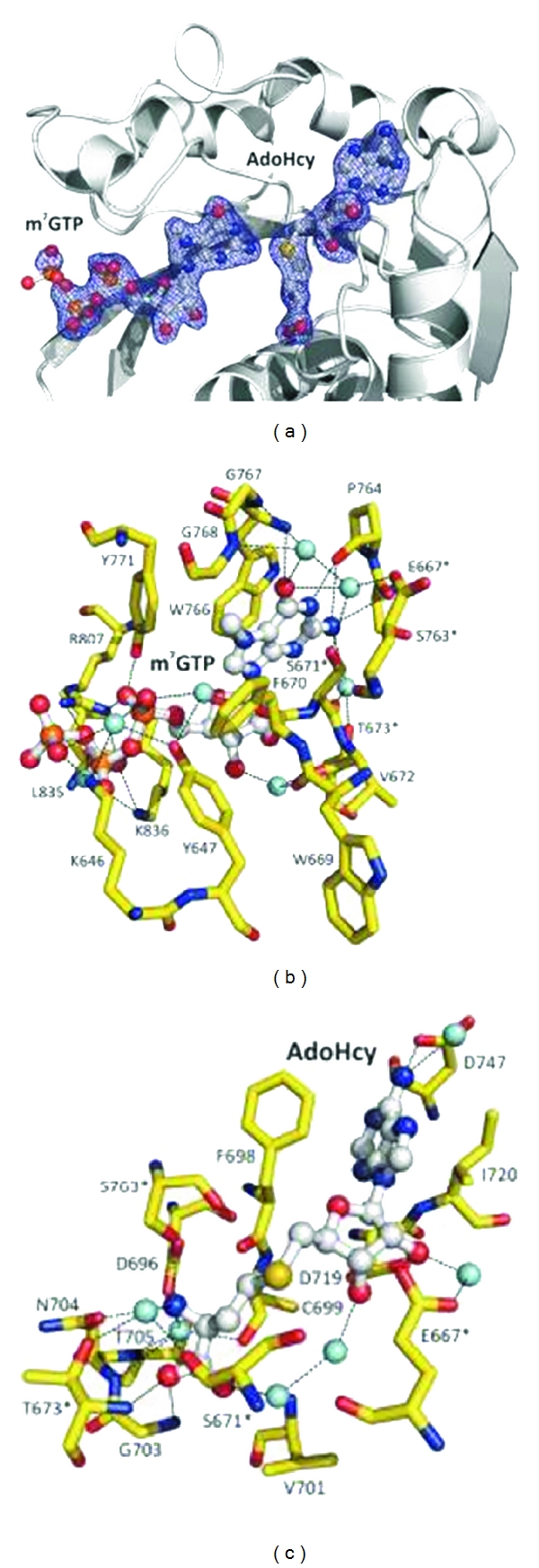
Crystal structure of hTgs1 [90]. The crystal structure of hTgs1 with substrate m^7^GTP and AdoHcy (at the site for AdoMet). The m^7^G is stacked between tryptophan 766 and serine 671 [90]. (a) Relative orientation of substrates m^7^GTP and AdoHcy at binding pockets. (b) Detailed view of the binding pocket for m^7^GTP (shown W766 and S671). (c) Detailed view of the binding pocket for AdoHcy.

The human Tgs1 (618–853) has been crystallized for structural analysis. The one monomer consists of 11 *α*-helices and 7 *β*-strands. It is composed of 2 domains, the core domain (Glu675-Asp844) and N-terminal extension (Leu34-Ser671) connected by 3 amino acids—Val672, Thr673, and Ser674. The core domain consists of 7-*β*-strands in topology of   *β*6↑*β*7 ↓ *β*5↑*β*4↑*β*1↑*β*2↑*β*3↑ with a classical class 1 methyltransferase fold resembling the Rossmann-fold AdoMet-dependent methyltransferase superfamily [90]. The N-terminal *α*-helices form a separate small globular subdomain involved in recognition and binding of both substrates. The residues Glu667 and Phe670 in motif X as well as Pro765, Trp 766, and Pro769 in motif IV are in proximity permitting the top of their binding clefts to be close together. Tryptophan 766 and m^7^G are stacked in a coplanar manner with a 3.2 Å distance providing a tight *π*-*π* interaction between them (Figure 34). 

**Figure 35:**
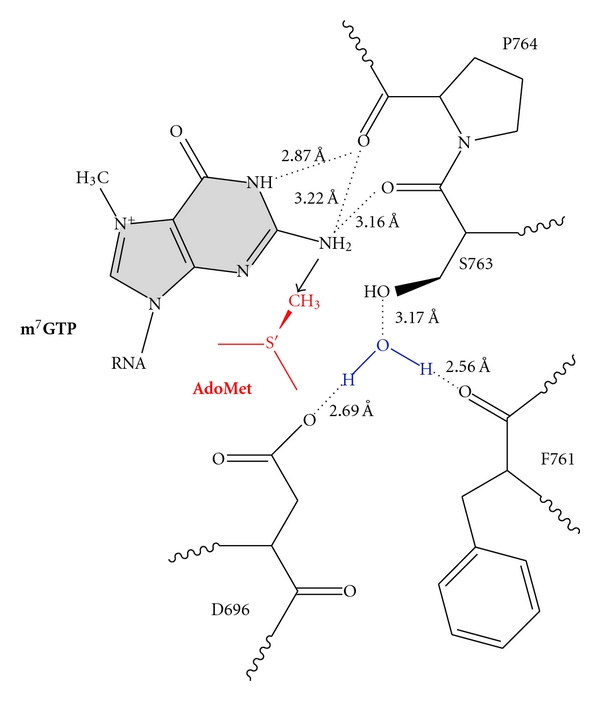
The active site of hTgs1 [90]. Proposed mechanism of methyltransferase activity of hTgs1 is shown. The AdoMet methyl group is in close proximity to N2 position of m^7^GTP. The prerequisite as a substrate for hTgs1 is m^7^G moiety.

The catalytic mechanism of methylation is by an S_n_2 substitution reaction. The N2 of m^7^G does the nucleophilic attack on an activated methyl group of the AdoMet (Figure 35).

 Dimethylation is not processive. After formation of m_2_^2,7^G both products (m_2_^2,7^G and AdoHcy) dissociate from the enzyme. Tgs1 can use m_2_^2,7^G as a substrate, and newly bound AdoMet can methylate at the same position by the same mechanism to form the m_3_^2,2,7^G cap structure.

### 12.2. Drosophila Tgs1

In Drosophila melanogaster, DTL (Drosophila TAT-like) has been reported to exhibit trimethylguanosine cap formation activity for both U2 and U4 snRNAs. The mRNA for the protein Tgs1 is polycistronic and 2,600-nucleotide long with upper and downstream ORFs (open reading frames). The uORF is 80 bp from the transcription start site and has coding capacity for a 178 amino acid protein while dORF is 538 bp from the 5′ end and produces a 60 kDa protein (491 amino acids). The two cistrons are overlapped by 76 bp. Mutational analysis indicates that both the uORF and dORF regions are required for viability. The putative product of uORF contains periodic Leu residues, but there is no evidence that this region is translated at any time during Drosophila development. The protein from dORF contains an Arg-rich motif KKKRRQRQI similar to the RNA binding motif RKKRRQRRR in HIV TAT. This protein is localized in the nucleus and responsible for trimethylation of U2 and U4 snRNAs [111].

### 12.3. Yeast Tgs1

In yeast *S. cerevisiae*, Tgs1 is in the nucleolus and U3 RNA is also in the nucleolus. In the absence of Tgs1, the pre-U3 RNA was found within the nucleolar body and U1 RNA was retained in the nucleolus. *S. cevisiae, S. pombe,* and *Giardia lamblia* Tgs1 can methylate m^7^GTP, m^7^GDP, and m^7^GpppA as substrates without preassembly of snRNP containing Sm proteins. The Tgs1 of S pombe is 239-amino-acid long and m^7^G is the pre-requisite for this reaction [112].

### 12.4. The G. lamblia Tgs1 and Tgs2

The *lamblia* has 2 enzymes, Tgs1 and Tgs2. Tgs 1 is not a processive enzyme but distributive and produces m_3_^2,2,7^G in excess of AdoMet and enzyme. However, Tgs2 produces only m_2_^2.7^G, and some *G. lamblia* RNAs contain dimethylG caps. The G lamblia Tgs1 has 300 amino acids and Tgs2 is 258 amino acids long. They all have landmark motifs for Ado-Met-dependent methyltransferase motifs [113].

## 13. Parasite Capping Enzyme *(Trypanosoma brucei)*

The parasite *Trypanosoma brucei* SL RNA (splicing leader) has the biggest 5′ oligonucleotide, type IV, of m^7^Gpppm_2_^6,6^AmpAmpCmpm^3^UmpAp [114, 115]. Enzymes involved in the synthesis of this cap structure are TbCgm1, TbCet1, TbMTr1(cap1 2′OMTase), TbMTr2/TbCom1/TbMT48(cap2 2′OMTase), TbMTr3/TbMT57(cap3 2′OMTase). However, m_2_^6,6^A and m^3^Um methylating enzymes have not been identified as yet [115]. 

### 13.1. TbCgm1 (*T. brucei* Cap Guanylyltransferase Methyltransferase 1)

There exist two enzyme systems for 5′ cap formation. The first is the system composed of separate independent enzymes which are TbCet1 (*Trypanosoma brucei* triphosphatase, 253 amino acids), TbCe1 (*Trypanosoma brucei* guanylyltransferase, 586 amino acids), and TbCmt1 (*Trypanosoma brucei* m^7^G Cap methyltransferase 1, 324 amino acids). The second is a set of fused enzymes possessing dual activities. It is TbCgm1 (*Trypanosoma brucei* cap guanylyltransferase and methyltransferase 1) that has 1050 amino acids [116] with dual activities of guanylyltransferase and guanine N-7 methyltransferase [117]. The TbCe1 guanylyltransferase has 250 amino acids at its N-terminal region which is not found in fungal or metazoan guanylyltransferase and has homology with the phosphate binding loop found in ATP- and GTP-binding proteins [118]. Silencing TbCe1 and TbCmt1 had no effect on parasite growth or SL RNA capping, but TbCgm1 was essential for parasite growth and silencing TbCgm1 increased the amount of uncapped SL RNA. The protein TbCgm1 has guanylyltransferase activity in N-terminal 1–567 amino acids and methyltransferase activity in C-terminal 717–1050 amino acids. The N-terminal guanylyltransferase portion contains 6 colinear guanylyltransferase motifs: I(KADGTR), III(FVVDAELM), IIIa(LIGCFDVFRYVI), IV(DGFIF), V(QLXWKWPSMLSVD), and VI(WSIERLRNDK). The C-terminal methyltransferase portion contains regions homologous to m^7^G methyltransferase from *T. cruzi* and *L. major* [117].

### 13.2. Cap Methylating Enzymes: TbMTr1, TbMTr2(TbCom1/TbMT48), and TbMTr3(TbMT57)

They contain a K^95^-D^207^-K^248^-E^285^ tetrad critical for AdoMet-dependent methyltransferase and can convert cap type 0 of *Trypanosoma* SL RNA and U1 snRNA into type 1 cap [115]. The KDKE mediates S_*n*_2 type transfer of methyl groups that involve 2′-OH deprotonation. The U1 snRNA 2′-O-methylation takes place before Sm protein binding to the RNA and it is prerequisite for the dimethylation at the N2 position to make m_3_^2,2,7^GpppAm cap structures. Other m_3_^2,2,7^G cap-containing snRNAs such as U2, U-snRNA B (U3 snRNA homolog), and U4 snRNAs were reported to be synthesized by RNA polymerase III in *Trypanosomes*.

The TbMTr2 and TbMTr3 are responsible for second and third nucleotides 2′-O-methylations. The enzymes that perform m_2_^6,6^A, m^3^U base methylations, and fourth nucleotide 2′-O-methylating enzymes are not known yet.

## 14. Transport of Mature RNAs

**Figure 36:**
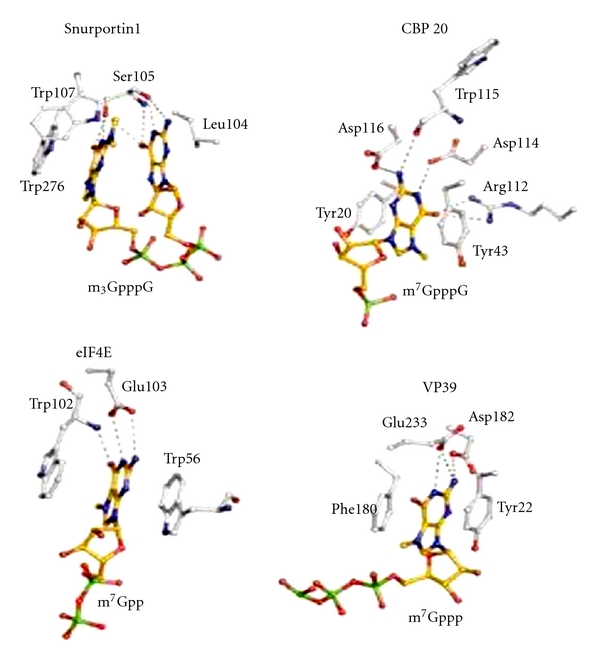
The comparison between m_3_^2,2,7^G binding mode to snurportin1 and m^7^G cap binding mode to CBP 20, eIF4E and VP39 [91]. The m_3_^2,2,7^G is stacked between tryptophan 107 and penultimate nucleotide G of cap core m_3_^2,2,7^GpppG.

**Figure 37:**
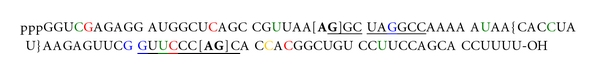
The sequence of 4.5S RNA I (Novikoff hepatoma cell nuclei).

The snurportin1 is a specific trimethyl G cap binding protein with an importin *β* binding site at its N-terminus (amino acids 1–65) and trimethyl G cap binding site at amino acids 95–300 forming a cap binding pocket. This protein has more resemblance to mRNA guanylyltransferase. The snurportin 1 binds the trimethyl G cap forming *π*-stacking with tryptophan 276 and the penultimate purine nucleotide G (Figure 36). The tryptophan 107 is in close proximity to dimethylamine of N2 G suggesting a cation-*π* interaction and has a role in discriminating between m^7^G cap and m_3_^2,2,7^G cap [91].

## 15. Tgs 1 Interacting Proteins

Genetic and biochemical analysis of Tgs1 interacting proteins reveals a wide range of proteins involved in RNA metabolism. It interacts with proteins in the transcriptional apparatus, RNA end processing and decay, spliceosomal assembly and RNA modifying factors (Table 12).

Structurally, it is distinct from the m^7^G cap, and the specificity of binding proteins may determine the precision of its functional role in the RNP complex. The m_3_^2,2,7^G cap structures are present only in nuclear snRNAs and snoRNAs which confer the function within the nucleus in transcription, splicing, modification, processing, and maturation of different RNA species.

## 16. Conclusion

### 16.1. General Consideration

In the present postgenomic era, study of the structure and function of noncoding RNAs is supremely important. It is estimated that ncRNAs are probably involved in all aspects of cell metabolism. Therefore, RNA-based information will contribute greatly to understanding various cell metabolisms. In the process of exploring ncRNAs, there may be many surprises awaiting us.

They may include

1. new species of RNA,
2. new mechanism of RNA processing,
3. new mechanism of transcription,
4. new disease caused by RNAs with pathogenic sequences,
5. new function for ncRNA.

### 16.2. The Problem of Unknown Modified Nucleotides

In the process of oligonucleotides cataloging, it is natural that an examination of base composition will reveal modified nucleotides or nucleosides in addition to unmodified standard nucleotides or nucleosides. In routine work, identification of modifications can be readily made by two-dimensional paper chromatography for nucleotides or thin layer chromatography for nucleosides. However, there may be an occasion where chromatographic identification is not sufficient. Of course, it is best to have collaboration with outside specialists. For the sake of structural microanalysis, it is highly recommendable to determine molecular weight of the unknown nucleotide or nucleoside by mass spectrometry [119]. The required quantity is approximately 5 *μ*g/nt where chromatographic identification of isotopically labeled sample requires 0.5 *μ*g/nt. A difficulty may be confronted with purine bases that are fused to an imidazole ring (Queuosine) which is not suited for mass spectrometry. It is convenient to probe chemical complexity based on mass. The detailed analysis may require an unpredictably large amount of samples. There are 135 modified nucleosides listed, among which 6 nucleosides are not thoroughly identified [1].

### 16.3. Significance of Sequence Work

Past sequence work has permeated numerous significant areas of research providing a better understanding of cellular metabolism. The information obtained thus far is RNA-based information which is not seen in DNA, proteins, and others. As sequence work continues to make enormous progress, the postgenomic era will shape the direction of research in the area of molecular mechanisms of RNA metabolism. They are briefly as follows.

In RNA maturation, knowledge of structural modifications is necessary to discern between various mechanistic options. For example, there are two molecular mechanisms mediated by catalysis. One is mediated by RNA enzymes (snRNAs and snoRNAs) involved in splicing of pre-mRNA and processing of pre-rRNA. The other is protein enzymes involved in 5′ cap formation. Currently, the higher order structural analysis is in progress. There is a need to elucidate the details of molecular mechanisms. 

Along with the study of splicing physiology, splicing pathology is making significant progress. Aberrant modifications can generate disease causing alterations in structure. The aberrations cause problems in reading both genetic codes and splicing codes. Studying the regulation of alternative splicing will clarify the selective rules in intron removal and pathogenic rules in splicing code. From these studies, corrective strategy will evolve. The present sequence work is engaged in definition of ncRNAs diversity and their functional roles [120]. Since it is suggested that ncRNAs are involved in all aspects of regulations in cell metabolism, there may be opportunities to study various paths in cell metabolism, not limited to transcriptional and posttranscriptional events. It is this gigantic task, to reevaluate the genomic work, that holds excitement and promise.

##  Abbreviations Used in Table 4

**Short Noncoding RNA (Usually Shorter Than tRNA and Some Are Longer but Excluding snRNA Such As U1–U13)**

miRNA: MicroRNA (imperfect base pairing)
siRNA: Small interfering RNA (perfect base pairing)
  tasiRNA: Transacting small interfering RNA
  natsiRNA: Natural antisense transcribed small interfering RNA
piRNA: PIWI interacting RNA (RNA precipitated by PIWI protein antibody)
  rasi RNA/pitRNA: Repeat-associated small interfering RNA/pi-target RNA
PARs: promoter associated RNAs
  PROMTs: Promoter upstream transcripts (sense and antisense transcript)
  PASRs: Promoter-associated small RNAs
  TSSa-RNAs: Transcription-start-site-associated RNAs
  tiRNAs: Transcription initiation RNAs
MSY-RNA: MSY2-associated RNAs (MSY; Y chromosome male-specific protein)
snoRNA: Small nucleolar RNA (C/D box RNA, H/ACA RNA)
sdRNA: sno-derived RNAs
moRNA: MicroRNA-offset RNAs
tel-sRNA: Telomere small RNAs
crasiRNA: Centrosome-associated small interfering RNAs
hsRNA: Heterochromatin small RNA or hairpin small RNA
scaRNAs: Small Cajal-body-associated RNAs
Y RNAs: Cytoplasmic small RNA Y1, Y3, Y4, and Y5
tRNA-derived RNAs: Small RNA processed from tRNA by RNase (angiogenin)
Alu/SINE RNA: Alu restriction enzyme cleaved repeat gene transcript/short interspersed nucleotide element RNA.

**Lnc RNA: Long Noncoding RNA (~0.5 to 100 kb)**

**(1) Specific Long Noncoding RNA**

TR/TERC: Telomerase RNA/telomerase RNA component
NEAT RNAs: Nuclear enriched abundant transcript 1 RNAs
  NEAT1v-1: NEAT1 variant 1
  NEAT1v-2: NEAT1 variant 2
  NEAT2/MALAT1: Metastasis associated in lung adenocarcinoma transcript 1
PINC RNA: Pregnancy-induced noncoding RNA
DD3/PCA3: Prostate-cancer-associated RNA 3
PCGEM1: Prostate cancer gene expression marker 1
SPRY4-1T1: Sprouty homolog 4 gene transcript 1 (melanoma specific).

**(2) Imprinting-Associated lncRNAs**

xiRNAs: X chromosome inactivating RNAs
  Xist: X chromosome inactivating sense RNA
  Tsix: Antisense transcript of Xist
  RepA: Repeat A RNA
AIR RNA: Igf2r imprinting region RNA
H19: Igf2 imprinting region RNA
KCNQ1ot1: Antisense RNA from intron 10 of Kcng1 gene imprinting region.

**(3) Regulatory lncRNAs**

HOTAIR: Homeogene inactivating RNA
BORG: BMP/OP-responsive-gene-associated RNA
CTN RNA: Cationic amino acid transporter protein coding region RNA
ANRIL RNA: Antisense noncoding RNA in INK4 locus.

**(4) Gene-Recombination-Associated lncRNA**

LINE: long interspersed nucleotide element
CSR-RNA: Immunoglobulin class switch recombination region RNA.

**(5) Satellite DNA Transcripts**

 

##  Abbreviations for Table 12

Mud: Mutant U1 die
RES complex: Heterotrimeric RNA retention and splicing complex composed of Bud13, Ist3/Snu17, and Pml1
Swt21: Synthetic with Tgs1 number 21
TRAMP complex (Trf4, Air2, Mtr4p polyadenylation complex): Interacts with exosome in the nucleus and involved in 3′ end processing of rRNA, snoRNA, and U1, U4, and U5 snRNA;
  Trf4 or Trf5: poly(A) polymerase(PAP);
  Mtr4: RNA helicase;
  Air1 or Air2: Zinc knuckle protein
Cbf5(YLR175W): Centromere binding factor;
  Pseudouridine synthase catalytic subunit of box H/ACA snoRNP complex
PIMT: PRIP-interacting protein with methyltransferase domain, PIMT is a Tgs1 (trimethylguanosine synthase 1) cloned from human liver cDNA library
PRIP: PPAR interacting protein
PPAR: Peroxisome proliferator-activated receptor
PBP: PPAR binding protein.

## Figures and Tables

**Table 1:** Signature sequences and modifications of major snRNAs. The 5′ cap and 3′ nucleosides, base modified nucleosides, and alkali resistant oligonucleotides were determined by many methods described in the text. The table provides a summary of individual RNA characteristics of rat Novikoff hepatoma cells.

RNA	Subspecies	Localization	5′ End	3′ End	Modified Nucleotides
4.5S RNA	I	Extranucleolar nuclei	pppG	U-OH	
	II (U6)	Extranucleolar nuclei	mpppG	U-OH	3Ψ, m^6^A, m^2^G, AmA, AmG, AmGmC, CmC, CmA, CmCmU
	III	Extranucleolar nuclei	pA	Um	AmA, GmA, 2GmG, m^2^G, m^6^A, 3Ψ

5S RNA	I	Nucleoli, nuclei and cytoplasm	pppG	U-OH	
	II	Nucleoli, nuclei and cytoplasm	pppG	U-OH	
	III (U5)	Extranucleolar nuclei	m_3_^2,2,7^GpppAmUmAC	U-OH A-OH	UmU, GmC, 2Ψ

U1 RNA	U1a (5.8S RNA)	Nucleoli and cytoplasm	pC, pG	U-OH	UmG, GmC, Ψ
	U1b, U1c	Extranucleolar nuclei	m_3_^2,2,7^GpppAmUmAC	U-OH, G-OH	AmC, 2Ψ

U2 RNA		Extranucleolar nuclei	m_3_^2,2,7^GpppAmUmC	C-OH A-OH	GmGmC, GmG, GmA, m^6^AmG, CmΨ, UmA, CmU, 13Ψ, (m^6^A, m^2^G)

U3 RNA	U3a, U3b, U3c, U3d	Nucleoli	m_3_^2,2,7^GpppAmA(m)AG	A-OH, U-OH, C-OH	2Ψ

**Table 2:** Characteristics of RRM/RNP/RBD domain.

(1)	~90–100 amino acids domain and most abundant in vertebrates
(2)	Many RNA binding proteins contain more than one RRM
(3)	Contain 2 conserved RNP1 (RGQAFVIF in *β*3) and RNP2 (TIYINNL in *β*1) in 4 antiparallel *β*-sheets of *βαββαβ*-fold
(4)	Binds 2–8 nucleotides of RNA (2 in CBP20, nucleolin and 8 in U2B′′)
(5)	A typical RRM containing 4 nucleotide binding sites (UCAC)
(6)	3 conserved aromatic amino acids (Y, F, W, H or P) in central *β*-strands (2 in RNP1 of *β*3 and 1 in RNP 2 in *β*1)
(7)	2 RRMs in a protein are separated by small linker and provide a large RNA binding surface or RNA binding surface point away from each other
(8)	RNA bases are usually spread on the surface of protein domains while the RNA phosphates point away toward the solvent
(9)	Binding surface of the protein is primarily hydrophobic in order to maximize intermolecular contact with the bases of the RNA
(10)	Few intramolecular RNA stacking and many intermolecular stacking mediated by aromatic amino acids
(11)	RNA recognition is a two-step process, in which any RNA is attracted approximately equally well. However, if stacking and hydrogen-bond interactions that “lock” the interaction cannot be properly established, the complex redissociates quickly (large *k*_*off*⁡_), which results in overall weak affinity for RNA oligonucleotides of the wrong sequence
(12)	Many ssRNA binding proteins recognize RNA in the loop (stem-loop) better than in ssRNA (*k*_*on*⁡_ ~ 3 fold & *k*_*off*⁡_ ~ 590 fold, therefore, overall affinity ~2000 fold differences) due to higher entropy loss with ssRNA binding than stem-loop binding and stabilizing interactions of stem

**Table 3:** Frequency of stem loops in primary pre-mRNA transcripts. The simple stem loops with minimal 3 nucleotides in the loop and minimal 3 base pairs in the stem consisting of AU, GC, and GU pairs have been constructed with the aid of a computer [46]. The total number of nucleotides were divided by numbers of stem loops for frequency. The number of nucleotides in each loop and each stem and spacer were counted and averages were calculated. (1) Human insulin gene transcript: 1,430 nt. (2) Human HDHGT (25-hydroxyvitamin D3 1-*α*-hydroxylase gene transcript): 4,825 nt. (3) Human FMR1 (fragile mental retardation 1) gene transcript: 39,224 nt. (4) Chicken ovomucoid gene transcript: 6,067 nt.

Transcript	nt/loop	nt/stem	nt in spacer	Frequency
(1) Insulin	4.6	7.4	3.5	15.5
(2) HDHGT	5.8	7.0	4.9	17.6
(3) FMR1	5.0	6.8	3.4	15.3
(4) Ovomucoid	5.6	7.0	3.7	16.0

**Table 4:** Paradoxical characteristics of ncRNAs in humans and mice [50, 51]. The excessive number of transcripts than anticipated for 25,000 genes indicates that the ncRNAs which were not detected due to scarce abundance have been detected by more sensitive methods. Some of these characteristics are summarized.

	Human	Mouse
Gene Number	75,000, 84,000 or 140,000 (cDNA identified)	
Transcripts		181,000
Population	50% Poly-A RNAs (of 16% genome)	50% transcripts (of 62% genome) (35% from antisense strand)
Intron	30% genome	
Processing	Polyadenylation, 5′ cap, splicing, nucleotide modification	
Transcripts from	Intergenic, Intronic regions and antisense strand	
Short ncRNAs	miRNA, siRNA (tasiRNA, natsiRNA), piRNA, rasiRNA (pitRNA), PARs (PROMTs, PASRs, TSSa-RNAs, tiRNAs), MSY-RNA, snoRNA, sdRNA, moRNA, tel-sRNA, crasiRNA, hsRNA, scaRNAs, AluRNA, YRNA, tRNA-derived RNAs	
Long ncRNA (lncRNA) (0.5–100 kb)	Cancers, disorders in skin, heart, brain, cerebellum, and so forth. TR/TERC, NEAT RNA (NEAT1v-1, NEAT1v-2, NEAT2/MALAT1), PINC RNA, DD3/PCA3, PCGEM1, SPRY4-1T1, xiRNAs (Xist RNA, Tsix RNA, RepA RNA), AIR, H19, KCNQ1ot1, HOTAIR, BORG, CTN RNA, ANRIL RNA, LINE, CSR RNA, satellite DNA transcripts and so forth	
Function	Regulatory function in all aspects of metabolism [52]	

**Table 5:** Examples of modification. All RNA species including high- and low-molecular-weight RNAs have their own signature sequences and modifications.

RNA	Sequence and signature modification
hnRNAs (Exons + Introns)	m^7^G cap, m^6^A, Poly-A, splicing codes
mRNAs (mainly exons)	m^7^G cap, m^6^A, Poly-A Editing (C→U & A→I)
tRNAs	TΨC, CCA, Many hypermodified bases
45S pre-rRNA	Repeated U sequences at 5′ end spacers
18S rRNA	m_2_^6^A, hypermodified m^1^acp^3^Ψ
28S rRNA	NmNmNmN, NmNmN
snRNAs, snoRNAs	m_3_^2,2,7^G cap, m*γ*G cap Types I, II, and III caps by ribose methylations
mRNA (Trypanosome)	m^7^Gpppm_2_^6,6^AmpAmpCmpm^3^UmpAp Insertion of repeated U sequence Deletion of U sequence

**Table 6:** General schemes of RNA sequencing. The direct and indirect methods of RNA sequencings are briefly outlined. The cDNA and DNA pathways are considered indirect methods.

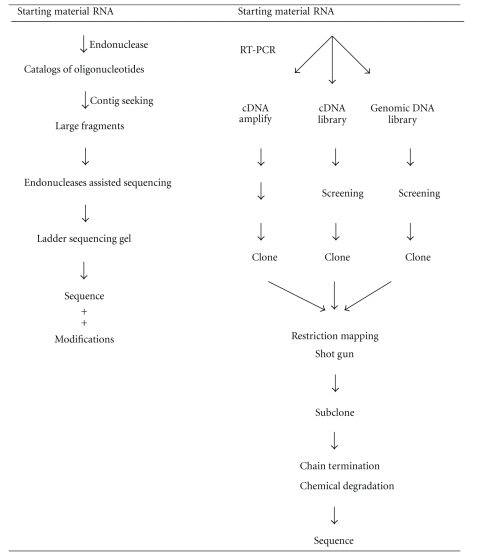

**Table 7:** Reagents and procedures required for sequencing.

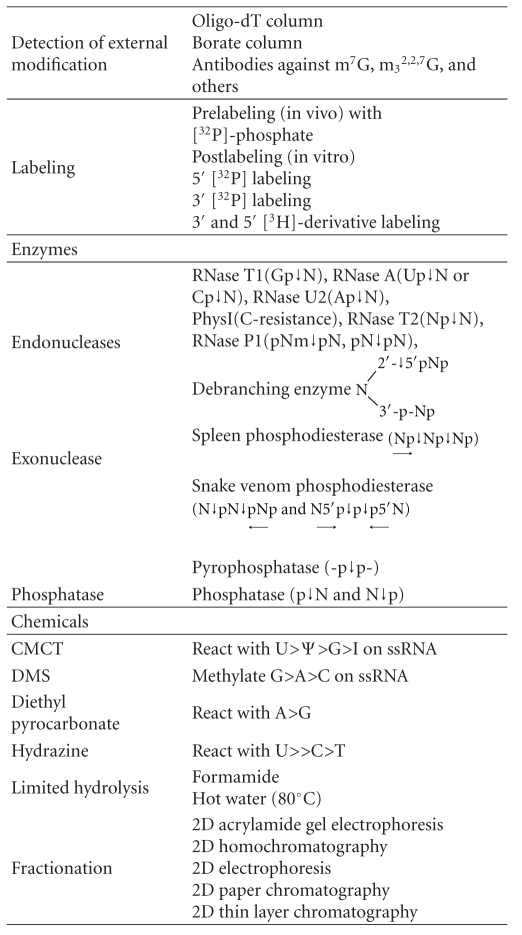

**Table 8:** Distribution of ESE, 5′ splice sites, branch sites, and 3′ splice sites. The ESE, 5′ splice site, branch site, and 3′ splice site in 4.5S RNA I and Alu elements in FMR1 gene transcript are screened by ESE finder (version 3) [13]. For this comparison, the number of motifs is calculated per 100 nucleotides. The motif patterns in Alu elements are all very much alike and the 4.5S RNA I resembles them. A difference is found in that 5′ splice sites in (+) Alu are more than in (−) Alu and 3′ splice sits are more in (−) Alu than in (+) Alu.

	SF2/ASF	SC35	SRp40	SRp55	Total	5′SS	BS	3′SS
Novikoff 4.5S RNA I (96 nt)	3.65	3.13	6.25	1.04	**14.07**	0	10.4	2.08
Human FMR1 Alu1(+) (252 nt)	4.96	7.54	6.35	1.59	20.44	3.97	9.13	5.56
Human FMR1 Alu4(+) (295 nt)	4.41	4.41	1.69	2.03	12.54	2.37	10.9	2.37
Human FMR1 Alu5(+) (246 nt)	5.69	5.28	4.47	1.22	16.66	4.47	8.54	2.44
Human FMR1 Alu7(+) (290 nt)	6.21	2.41	3.10	0.34	12.06	3.79	10.3	2.07
Human FRM1 Alu8(+) (288 nt)	5.21	3.47	3.82	0.69	13.19	3.47	8.68	2.43
(+) Alu Av.	5.30	4.62	3.89	1.17	**14.98**	**3.61**	9.51	2.97
Human FMR1 Alu2(−) (298 nt)	3.69	6.04	5.03	1.34	16.10	0.67	14.1	4.70
Human FMR1 Alu3(−) (285 nt)	4.92	7.02	5.61	2.11	19.66	3.16	13.7	4.56
Human FMR1 Alu6(−) (290 nt)	4.31	5.52	4.48	1.38	15.69	2.76	13.5	4.48
(−) Alu Av.	4.31	6.19	5.04	1.61	**17.15**	2.20	13.8	**4.58**
Human FMR1 Total Alu(+&−) Av.	4.93	5.21	4.32	1.34	**15.80**	3.08	11.1	3.58

**Table 9:** The snRNA 2′ and 3′-OH labeling by NaIO_4_ oxidation and [^3^H]-KBH_4_ reduction [38]. The total 4–7S RNA from rat Novikoff hepatoma cell nuclei was labeled with [^3^H] by oxidation with NaIO_4_ followed by [^3^H]-KBH_4_ reduction (Figure 12). Individual RNA species were purified by gel electrophoresis (Figure 13). The RNA samples were hydrolyzed with 0.3 N KOH, and hydrolysates were chromatographed on whatman 3MM paper according to de Wachter and Fiers [55]. The radioactivities at the origin (22% for 5S RNA, 54.1% for U1 RNA, 49.7% for U2 RNA, and 50.6% for U3 RNA) represent % of total radioactivity applied and they represent the 5′ end labeling which was later elucidated by many enzymatic methods described in the text. The radioactivities moved by chromatography with standard nucleoside derivatives are the % of total in nucleosides derivatives. The A′ U′ G′ C′ represent trialcohol derivatives of nucleosides.

		3′ Nucleoside derivatives			
RNA Species	Radioactivity at origin (5′)	A′	U′	G′	C′
	%	%	%	%	%
4S RNA	10.9	89.0	3.2	3.9	3.8
4.5S RNA	15.8	11.2	79.7	4.0	5.1
4.5S RNA I	6.5	6.1	87.4	4.7	1.8
4.5S RNA II	30.9	13.1	80.2	4.7	2.4
5S RNA	22.0	11.4	75.5	6.0	7.0
U1 RNA	54.1	6.0	13.4	77.7	3.0
U2 RNA	49.7	61.5	6.0	4.3	28.2
U3 RNA	50.6	53.8	22.6	9.8	13.7

**Table 10:** The [^32^P]cpm radioactivity distribution in nucleotides and cap core produced by RNase P1 digestion of U1 5′ oligonucleotide [16]. The [^32^P]-labeled U1 5′ oligonucleotide obtained by digestion of U1 RNA with RNase T2, RNase U2, and alkaline phosphatase was treated with P_1_ nuclease which cleaves all phosphodiester bonds but not pyrophosphate bonds. The products were separated on a DEAE column (Figure 23). The radioactivity in peak a (mononucleotides pUm, pA) and peak b (cap core m_3_^2,2,7^GpppAm) were determined by Packard liquid scintillation spectrometer.

	Peak a (pUm, pA)	Peak b (m_3_^2,2,7^GpppAm)
U1 5′ cap	65,280	87,600

**Table 11:** The analysis of [^32^P]-labeled U2 RNA 5′ oligonucleotide [12]. The 5′ ends obtained from uniformly [^32^P]-labeled U2 RNA were digested with T1 RNase or RNase A and isolated by two-dimensional electrophoresis (cellulose acetate at pH 3.5 followed by DEAE paper electrophoresis). The 5′ oligonucleotides were digested with snake venom phosphodiesterase before and after removal of 3′ phosphate with bacterial alkaline phosphatase. The products were separated as in Figure 27. The radioactivity ratios are listed.

5′ fragment from U2 RNA	^32^P ratio in nucleotide							
	Pi	pUm	pAm	pm_3_^2,2,7^G	pC	pCp	pG	pGp
5′ oligo from T_1_ digestion	1.58	1.33	0.91	0.90	1			1.48
Alkaline phosphatase digested T_1_5′ oligo	1.22	1.43	0.98	0.90	1		0.93	
5′ oligo from RNase A digestion	1.74	1.25	1	1.08		1.50		
Alkaline phosphatase digested A 5′ oligo	0.93	1.45	0.94	0.77	1			

**Table 12:** Tgs1 interacting proteins [70–72]. The Tgs1 (trimethylguanosine synthase 1) interacting proteins are listed. It is interacting with proteins involved in many aspects of RNA metabolism such as transcription, spliceosome assembly, maturation, and modification.

Tgs interacting proteins [70]	
Transcription apparatus (regulators of RNA polymerase II transcription) (1) Rpn4 (TF; Proteasome subunits and U2 snRNA gene) (2) Spt3 (SAGA complex) (3) Srb2 (mediator complex) (4) Soh1 (Med31; mediator complex) (5) Swr1 (ATPase; binds to 5′ end of yeast transcription units) (6) Htz1(H2AZ) (binds to 5′ end of yeast transcription units) (7) CBP (binds to human PIMT/Tgs1) (8) P300 (binds to PIMT/Tgs1) (9) PBP (binds to PIMT/Tgs1) (10) PRIP (binds to PIMT/Tgs1; [71])	Spliceosome assembly (1) Mud1 (yeast homolog of U1A) (2) Mud2 (yeast homolog of U2AF65) (3) Nam8 (Mud15) (homolog of TIA-1) (4) Brr1 (snRNP) (5) Lea1 (U2A; U2snRNP) (6) Ist3 (Snu17; U2 snRNP) (7) Isy1 (Ntc30; interacts with Prp19) (8) Cwc21 (component of Cef1 complex) (9) Bud13 (Cwc26) (Cef1 complex) (10) SMN (HeLa cell) [72]

RNA end processing and decay (1) Trf4 (Pap2; catalytic subunit of TRAMP) (2) Lsm1 (decapping complex) (3) Pat1 (decapping complex)	RNA modifying factors (1) SmB (snRNP; interacts with YNL187/Swt21) (2) SmDL (snRNP) (3) Cbf5 (snoRNP) (4) Nop58 (snoRNP) (5) Mrm (RNA 2′-O-methyltransferase)

**Table 13:** Cap variations of flanking nucleotide modifications.

	Trimethylguanosine cap	7-Methylguanosine cap
Type 0	m_3_^2,2,7^GpppN-	m^7^GpppN-
Type I	m_3_^2,2,7^GpppNmN-	m^7^GpppNmN-
Type II	m_3_^2,2,7^GpppNmNmN-	m^7^GpppNmNmN-
Type III	m_3_^2,2,7^GpppNmNmNmN-	m^7^GpppNmNmNmN-
Type IV	m_3_^2,2,7^GpppNmNmNmNmN-	m^7^GpppNmNmNmNmN-
